# Identification and bioinformatic characterization of a multidrug resistance associated protein (ABCC) gene in *Plasmodium berghei*

**DOI:** 10.1186/1475-2875-8-1

**Published:** 2009-01-02

**Authors:** María González-Pons, Ada C Szeto, Ricardo González-Méndez, Adelfa E Serrano

**Affiliations:** 1 Department of Microbiology, University of Puerto Rico School of Medicine, PO Box 365067, San Juan, PR 00936-5067, USA; 2 Department of Radiological Sciences, University of Puerto Rico School of Medicine, PO Box 365067, San Juan, PR 00936-5067, USA; 3 Department of Biochemistry, Universidad Central del Caribe, PO Box 60327, Bayamón, PR 00960-6032, USA

## Abstract

**Background:**

The ATP-binding cassette (ABC) superfamily is one of the largest evolutionarily conserved families of proteins. ABC proteins play key roles in cellular detoxification of endobiotics and xenobiotics. Overexpression of certain ABC proteins, among them the multidrug resistance associated protein (MRP), contributes to drug resistance in organisms ranging from human neoplastic cells to parasitic protozoa. In the present study, the *Plasmodium berghei mrp *gene (*pbmrp*) was partially characterized and the predicted protein was classified using bioinformatics in order to explore its putative involvement in drug resistance.

**Methods:**

The *pbmrp *gene from the *P. berghei *drug sensitive, N clone, was sequenced using a PCR strategy. Classification and domain organization of pbMRP were determined with bioinformatics. The *Plasmodium spp. *MRPs were aligned and analysed to study their conserved motifs and organization. Gene copy number and organization were determined via Southern blot analysis in both N clone and the chloroquine selected line, RC. Chromosomal Southern blots and RNase protection assays were employed to determine the chromosomal location and expression levels of *pbmrp *in blood stages.

**Results:**

The *pbmrp *gene is a single copy, intronless gene with a predicted open reading frame spanning 5820 nucleotides. Bioinformatic analyses show that this protein has distinctive features characteristic of the ABCC sub-family. Multiple sequence alignments reveal a high degree of conservation in the nucleotide binding and transmembrane domains within the MRPs from the *Plasmodium spp. *analysed. Expression of *pbmrp *was detected in asexual blood stages. Gene organization, copy number and mRNA expression was similar in both lines studied. A chromosomal translocation was observed in the chloroquine selected RC line, from chromosome 13/14 to chromosome 8, when compared to the drug sensitive N clone.

**Conclusion:**

In this study, the *pbmrp *gene was sequenced and classified as a member of the ABCC sub-family. Multiple sequence alignments reveal that this gene is homologous to the *Plasmodium y. yoelii *and *Plasmodium knowlesi mrp*, and the *Plasmodium vivax *and *Plasmodium falciparum mrp2 *genes. There were no differences in gene organization, copy number, or mRNA expression between N clone and the RC line, but a chromosomal translocation of *pbmrp *from chromosome 13/14 to chromosome 8 was detected in RC.

## Background

The ATP-binding cassette (ABC) superfamily is one of the largest evolutionarily-conserved families of protein transporters. ABC proteins play key roles in cellular detoxification of xeno- and endobiotics. Overexpression of certain ABC proteins, among them the multidrug resistance protein (MDR) and the multidrug resistance associated proteins (MRPs), contribute to drug resistance in a variety organisms ranging from parasitic protozoa to human neoplastic cells.

**Figure 1:**
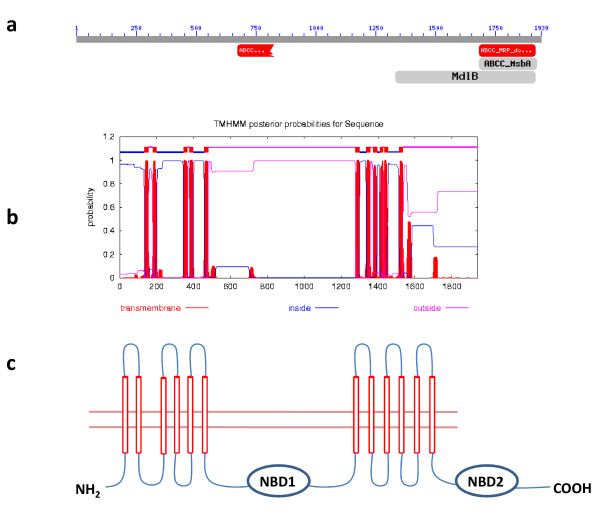
**Classification and structural organization of pbMRP**. (A) Conserved domain database summary view of the domain model identifying ABCC specific NBDs within pbMRP. These domains are depicted by red boxes under the corresponding regions within the predicted protein sequence. (B) Graphical representation the pbMRP predicted topology generated by the TMHMM server. Results were also confirmed using the DAS server transmembrane prediction. Predicted transmembrane helices are depicted in red. (C) Diagram of pbMRP embedded in the membrane. The protein contains two TMDs and two ATP-binding NBDs represented by red cylinders and blue circles, respectively.

Membrane transporters, such as the *Plasmodium falciparum *chloroquine resistant transporter (*pfcrt*) and the *Plasmodium falciparum mdr1*, which is a member of the ABC superfamily, have been identified as key contributors in decreasing susceptibility to several anti-malarial drugs [1-4]. Research to identify additional potential contributors to *Plasmodium *drug resistance has lead to the identification of new candidate transporter genes, some of which belong to the ABC transporter superfamily [5-8]. The ABC transporter superfamily is comprised of eight subfamilies in eukaryotes: ABCA, ABCB, ABCC, ABCD, ABCE, ABCF, ABCG, and ABCH. Proteins within this superfamily were classified based on the sequence and organization of their conserved nucleotide binding domains (NBD). Characteristic motifs within these NBDs are found in the majority of adenine nucleotide hydrolases: the Walker A and Walker B boxes, ABC signature motif, H (histidine) loop, D (aspartate) loop, and Q (glutamine) loop [9-13]. In general, functional ABC proteins contain two NBDs and two transmembrane domains (TMD) consisting of 6–11 transmembrane helices. Genes are organized either as full transporters containing two of each domain or half transporters with one of each (Figure 1c).

Members of the ABCC sub-family have been associated with drug resistance in organisms ranging from bacteria to man. This sub-family is comprised of a variety of proteins some of which have been designated as multidrug resistance associated proteins (MRPs). These proteins serve as primary active transporters of an array of structurally diverse compounds including organic anions such as glucuronide, glutathione (GSH), sulphate, drugs conjugated to GSH, and non-conjugated agents by GSH co-transport [14,15]. The human ABCC sub-family consists of 13 members, nine of which are transporters: MRP1, MRP2, MRP3, MRP4, MRP5, MRP6, MRP7, MRP8, and MRP9 [16,17]. The human MRP1 has been the most studied among the MRP proteins because of its ability to transport a broad range of anticancer drugs through cellular membranes mediated by GSH co-transport or by the export of GSH-drug conjugates [14,18]. In addition, MRPs have been associated with drug resistance in other organisms such as the heavy metal resistance protein MRP-1 in *Caenorhabditis elegans *[19], the GSH conjugate transporters AtMRP1, AtMRP2, and AtMRP3 in *Arabidopsis thaliana *[20-22], and yeast cadmium factor gene (*ycf*1) in *Saccharomyces cerevisiae *[23]. Several *mrp *orthologs have been identified and linked to drug resistance in protozoa such as *Trypanosoma brucei *and *Leishmania spp*. In *T. brucei*, reduced sensitivity to melarsoprol has been linked to the overexpression of the MRP homologue, *Tb*MRPA [24]. In *Leishmania*, the pentamidine resistance protein 1(PRP1/ABCC7) and MRPA (formerly known as P-glycoprotein A/ABCC3) were shown to confer pentamidine resistance and antimony resistance, respectively [25-29].

Homologues of these proteins have been identified in *Plasmodium spp.*, but there is no clear evidence linking this transporter to anti-malarial resistance. Expression of MRP homologues was reported in *P. berghei *and *P. yoelii *[7] as well as in *P. falciparum *[30]. The *P. falciparum mrp1 *gene is an intronless gene with highest mRNA expression in the late trophozoite and schizont stages. The *P. falciparum mrp2*, however, is mostly expressed in ring stages [31]. Protein expression of these genes has only been reported for pfMRP1, where it has only been detected in the schizont stage, except in the CQ resistant *P. falciparum *strain, FAC8, where it was also detected in the trophozoite stage [30]. Mutations within this gene have been associated with anti-malarial resistance in clinical isolates [5,6,32].

In this study, the *pbmrp *gene was partially characterized using bioinformatics and molecular biology methods. We sequenced *pbmrp *and classified it as an ABCC sub-family member. Multiple sequence alignments with MRPs from *P. y. yoelii, P. vivax, P. knowlesi *and *P. falciparum *reveal a high degree of conservation within transmembrane and nucleotide binding domains. Bioinformatic analyses indicate that pbMRP is homologous to a *Plasmodium spp. *MRP2 gene. In addition, the gene copy number, structural organization, chromosomal localization and mRNA expression levels of the *pbmrp *gene were determined in drug sensitive (N clone) and the drug resistant derived line RC which was selected under CQ pressure and displays the multidrug resistance phenotype.

## Methods

### *Plasmodium berghei *lines and maintenance

Random-bred Swiss albino female mice were infected intravenously with the *P. berghei *drug sensitive N clone or the RC line which was selected under CQ pressure and displays the multidrug resistant phenotype. These lines were kindly provided by Wallace Peters [33,34]. Mice infected with the RC line were dosed once with CQ 1 hr after infection in order to maintain drug selection pressure [35]. Platelets and white blood cells were removed from the infected blood by glass bead and CF-11 cellulose columns, respectively. Infected red blood cells (IRBCs) were differentially lysed with 0.15% saponin [36] and free parasites were collected.

### Nucleic acid extraction

Plasmodium *berghei *DNA was isolated by phenol/chloroform extraction [37]. Total parasite RNA was extracted using RNA STAT-60 (Tel-Test Inc.) according to manufacturer's specifications. Chromosome blocks were prepared as described by Serrano *et al *[38].

### PCR amplification, cloning and sequencing of the *P. berghei mrp*

Plasmodium *berghei *genomic DNA from the N clone was subjected to PCR amplification using primers designed based on the *mrp *gene identified in *P. y. yoelii *[7] (Additional file 1). Amplification of *pbmrp *fragments was carried out under standard conditions. Amplified products were cloned into pGEM^®^-T-EASY (Promega) plasmids according to the manufacturer's instructions. Purified clones were sequenced using the Applied Biosystems Big Dye Terminator V3.0 sequencing chemistry (Davis Sequencing Inc., CA). Every position of the putative open reading frame for the *pbmrp *gene was sequenced at least twice in each direction.

### Bioinformatic analyses

The *pbmrp *gene was assembled by aligning the overlapping translated sequence of the *P. berghei *cloned PCR products along the translated *P. y. yoelii mrp *gene [GenBank: XP_725434]. Once the entire putative open reading frame (ORF) was assembled, the predicted amino acid sequence was analysed to identify conserved motifs using the InterProScan sequence search tool [39] and the Conserved Domain Search service [40]. The presence of internal transmembrane domains and their organization was predicted using the TMHMM v. 2.0 prediction server [41] and the DAS transmembrane prediction server [42]. *Plasmodium mrp *sequences were retrieved by sequence similarity searches using the *pbmrp *translated sequence from the PlasmoDB ver. 5.4 [43] and the BLAST search tool hosted at PlasmoDB and at NCBI [44,45]. To obtain the closest human homologue, we performed sequence similarity searches against human genome protein database using the *P. berghei mrp *predicted protein sequence.

The following sequences were recovered and used for subsequent sequence analyses: *P. falciparum mrp1 *[GenBank: ABV24500], *P. falciparum mrp2 *[GenBank: ABV24501], *P. vivax mrp1 *[GenBank: XP_001612680], *P. vivax mrp2 *[GenBank: XP_001617379]*, P. knowlesi mrp *[GenBank: CAQ42240], *P. berghei mrp *[GenBank; AAS46595], and *P. y. yoelii mrp *[GenBank: XP_725434]. Multiple sequence alignments and analyses were carried out using the ClustalW program hosted at the European Bioinformatics Institute [46]. Alignments were visualized by using GeneDoc (provided by the Pittsburg Supercomputing Center) [47]. The phylogenetic analysis of the MRPs from *Plasmodium *was performed as follows. The predicted protein sequences for the MRP genes from *Plasmodium *listed in Table 1 and the MRP2 from *Homo sapiens *[GenBank: CAB45309] (which was used as outgroup for placement of the root) were retrieved from NCBI in FASTA format. The multiple sequence alignment was done with ClustalW as described above. The alignment was submitted to the GBlocks server to trim the sections of large variability that would affect the phylogenetic analysis [48]. The GBlocks analysis was carried out using the less restrictive settings of smaller blocks and permitting gaps within the blocks. The suggested multiple sequence alignment, which retained approximately 49% of the alignment positions, was taken. The phylogenetic analysis was done using the PHYLIP suite of programs [49]. The program SEQBOOT was used to generate a bootstrapped data set of 500 replicates. The program PROTDIST was used to generate the distance matrices for the analysis. The rooted phylogenetic trees were constructed according to the program NEIGHBOR using the neighbor-joining algorithm [50]. The program CONSENSE was used to build a rooted consensus tree using an extended majority rule.

**Table 1:** *Plasmodium *MRP predicted protein *s*equence similarity to *pbmrp *from the *Plasmodium berghei *N Clone line

Gene*	**GenBank accession number**	**Nucleotide length (nt)**	**Predicted protein length (aa)**	**Percent sequence identity to *pbmrp***	**Percent amino acid similarity to *pbmrp***	**E value**
pyMRP	XP_725434	5928	1976	82%	88%	0
pkMRP	CAQ42240	6051	2016	36%	54%	0
pvMRP2	XP_001617379	6072	2024	36%	53%	0
pfMRP2	ABV24501	6327	2108	36%	56%	0
pvMRP1	XP_001612680	5181	1727	32%	51%	2.3e^-273^
pfMRP1	ABV24500	5469	1822	32%	53%	5.9e^-252^

* P. y. yoelii MRP (pyMRP), *P. knowlesi *MRP (pkMRP) *P. vivax *MRP2 (pvMRP2), *P. falciparum *MRP2 (pfMRP2), *P. vivax *MRP1 (pvMRP1), *P. falciparum *MRP1 (pfMRP1)

### Southern blot analysis

Southern blots and subsequent hybridizations were performed as described by Gervais *et al*., 1999 [51]. Genomic DNA (gDNA) from the *P. berghei *lines, N clone and RC, were digested with HindIII, EcoRI, or EcoRV. Membranes were hybridized with 5' and 3' *pbmrp *specific probes which were amplified with the following primer combinations: *pbmrp *5' (Forward primer-TAATATAGATAAAAATGAGGGGG and Reverse primer-AAAGTGCATAACAGTTACTTCC) and *pbmrp *3' (Forward primer-TACAATAGTTATGGCAATATTAG and Reverse primer-CATCAATAATTTCTTATCAGA). Hybridizations were carried out at 65°C in 2× sodium chloride/sodium citrate solution (SSC) with 0.1% sodium dodecyl sulphate (SDS).

### Chromosomal blot and localization

*Plasmodium berghei *(N clone and RC) chromosomes were separated by pulse-field gel electrophoresis [52,38]. To optimize separation (BioRad CHEF DRII) running conditions were modified as follows: 125 V, 120-s pulses for 24 h and 300-s pulses for 24 h at 14°C. Chromosomes were blotted onto a Zeta Probe membrane (BioRad) and hybridized at 65°C in 2× SSC/0.1% SDS with a radiolabeled *P. berghei mrp *specific 3' probe. Subsequently, the membranes were stripped and hybridized to known *P. berghei *chromosome markers: a chromosome 13/14 probe (probe 9.45 kindly provided by Dr. Marta Ponzi) [52] or a chromosome 8 probe directed to a known gene previously localized to this chromosome, the *P. berghei *γ-glutamylcysteine synthetase gene (*pbggcs*) [53,54].

### RNase protection assay (RPA)

Alpha-^32^P UTP labelled riboprobes for *pbmrp *and *β-tubulin *(*βtub*, used as a normalizing control) were synthesized *in vitro *by antisense transcription using the T7 RNA polymerase (Maxiscript^® ^SP6/T7 Kit, Ambion). Riboprobes were co-precipitated with dilutions of total RNA from the *P. berghei *sensitive N-clone or the drug resistant derived line RC. RPA was performed using the RPAIII™ system (Ambion, Austin, Texas) according to the manufacturer's instructions. Protected RNA hybrids were resolved on denaturing acrylamide gels which were subsequently exposed to autoradiography films. Autoradiograms were scanned and analysed using Quantity One 1-D Analysis Software (Bio-Rad, v. 4.4). Ratios of the densities of the normalized *pbmrp *signals were subsequently normalized to the drug sensitive N-clone to estimate mRNA expression levels in RC.

## Results

### Identification and characterization of an mrp homologue in *P. berghei*

**Figure 2:**
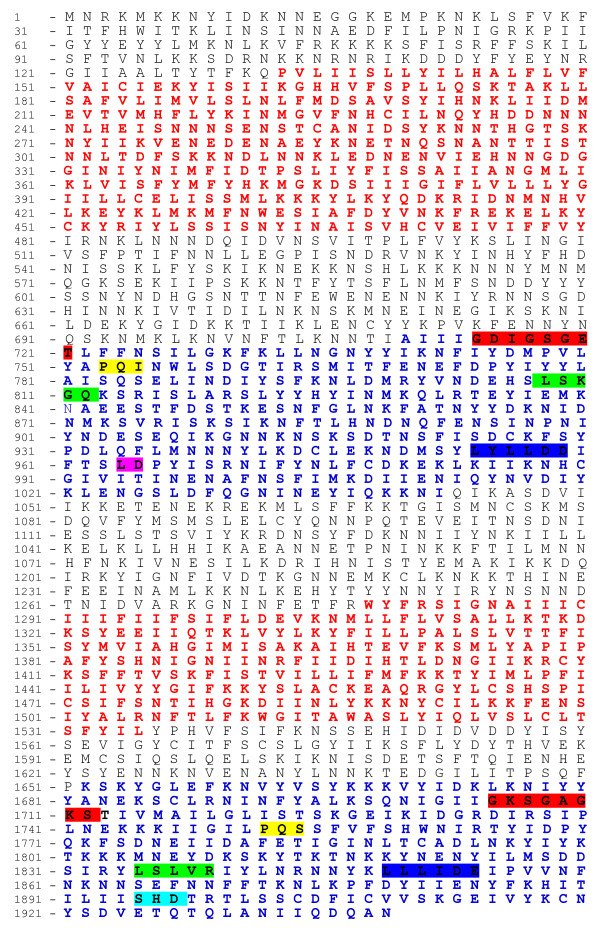
**Domain and motif organization in the pbMRP predicted protein sequence**. The pbMRP is comprised of 2 TMDs (red) and two NBDs (blue). Conserved sequence motifs within the NBDs are highlighted: the Walker A motif (red), the Q loop (yellow), the ABC motif (green), the Walker B motif (blue), the D loop (pink) and the H loop (turquoise).

An *mrp *homologue was identified in *P. berghei *using primers designed to PCR-amplify the *pbmrp *gene in the drug sensitive N clone. Sequence analysis of the *pbmrp *gene shows an open reading frame (ORF) of 5820 nucleotides. This single exon gene encodes a predicted protein of 1939 amino acids (Additional file 2). Based on the NBD amino acid sequence, the NCBI's Conserved Domain Database classified pbMRP as a member of the ABCC transporter sub-family (Figure 1a). This transporter possesses the general structural features associated with MRPs. Transmembrane helix predictions by both the DAS and TMHMM transmembrane prediction servers predict that pbMRP has 12 transmembrane helices organized into two TMDs and that both, the N-terminal and C-terminal, are intracellular. A model presenting domain organization of the predicted pbMRP amino acid sequence, which contains two TMDs and two NBDs, is shown in Figure 1c. Five of the six characteristic motifs associated with ABC NBDs were found within the NBDs of pbMRP (Figure 2). In NBD1, the Walker A, Q loop, ABC motif, Walker B and the D loop were identified, with a large variable insertion between the ABC motif and the Walker B motif. Within NBD2, the Walker A, Q loop, ABC motif, Walker B and the H loop were found. However, the H loop in NBD1 and the D loop within NBD2 were not found (Figure 2). Consensus motif sequences and the *Plasmodium spp. *motif sequences found within the NBDs are reported in Table 2.

**Table 2:** Motifs within *Plasmodium spp. *MRP nucleotide binding domains

**Nucleotide Binding Domain**	**Motif**	**Consensus Sequence**	**Consensus in *Plasmodium spp.***	**Location in pbMRP**
NBD1	Walker A	GxxxxGK [S, T]	G [D, N] [I, V]GSG [E, K]T	714–722
	Q loop	XQx	PQ [I, F]	753–755
	ABC motif	LSGGQ	LSKGQ	808–812
	Walker B	XILxDE	LYL [L, F]DD	954–959
	D loop	LD	LD	964–965
	H loop	XHx	Not identified	

NBD2	Walker A	GxxxxGK [S, T]	G [K, R]SGAGKS	1705–1712
	Q loop	XQx	PQS	1752–1754
	ABC motif	LSGGQ	L [S, A]LVR	1835–1839
	Walker B	XILxDE	L [L, V, I]LIDE	1849–1854
	D loop	LD	Not identified	
	H loop	XHx	[S, A]HD	1895–1897

**Figure 3:**
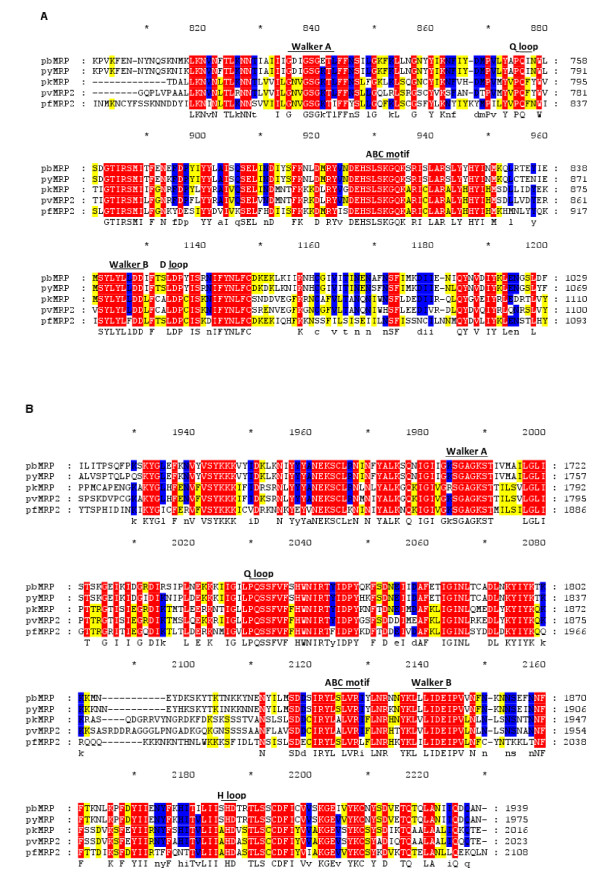
**Alignment of the of *Plasmodium spp*. MRP NBDs**. Alignment of predicted NBD1 (A) and NBD2 (B) amino acid sequences from *Plasmodium spp*. *mrp *genes were performed using the ClustalW algorithm with the Blosum matrix (all other parameters set as default). Amino acids with a 100% identity are shaded in red, 99–75% identity are in blue and 74–50% identity in yellow. Conserved Walker A, Q loop, the ABC motif, Walker B motifs as well D loop and H loop motifs are indicated above the corresponding sequence. Within the NBD1 there is a large non conserved region in NBD1 between the ABC motif and the Walker B and D loop containing region (from position 961 to 1110 in the multiple sequence alignment) which is not shown in this figure. For the complete multiple sequence alignment refer to Additional file 3.

**Figure 4:**
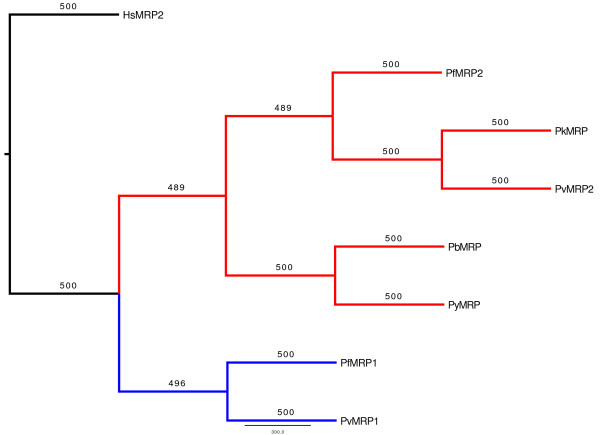
**Consensus rooted gene tree for *Plasmodium *multidrug-resistance associated proteins**. Consensus rooted gene tree was generated with the PHYLIP suite of programs using the neighbor-joining algorithm. The branch labels represent the bootstrap counts. The Homo sapiens MRP2 was used as an outgroup for root placement. MRPs have been designated as described in Table 1.

The predicted pbMRP amino acid sequence was used to identify homologues in other *Plasmodium *species by sequence similarity searches in PlasmoDB. A partial sequence was found in the *P. berghei *genome, two homologous genes in *P. falciparum *(*pfmrp1 *and *pfmrp2*), two in *P. vivax *(*pvmrp1 *and *pvmrp2*), one in *P. knowlesi *(*pkmrp*), and one in *P. y. yoelii *(*pymrp*). A multiple sequence alignment of the protein sequences shows that pbMRP has highest similarity with its homologue in the rodent malaria species, *P. y. yoelii *(Figure 3 and Additional file 3). Sequence identity between pbMRP and the MRPs from *Plasmodium spp*. ranges from 82–32% with the highest percent identity with pyMRP and the lowest with pfMRP1 (Table 1). BLAST results show that pbMRP is more closely related to pyMRP, pkMRP, pfMRP2 and pvMRP2 with expectation values reported as 0 although it also exhibits a high degree of relatedness to pfMRP1 and pvMRP1 (Table 1). Sequence similarity searches against the human genome protein database show that MRP2 is the closest homologue to pbMRP with an expectation value of 9e^-30^. The phylogenetic gene tree supports the results presented in Table 1 (Figure 4). The MRP1 subfamily and the MRP2 subfamily are clearly grouped into separate clusters indicating these are paralogous genes. The bootstrap values for all branches are above 97 percent indicating highly significant support for the clades seen in the gene tree.

### *pbmrp *gene organization in drug sensitive and resistant lines

**Figure 5:**
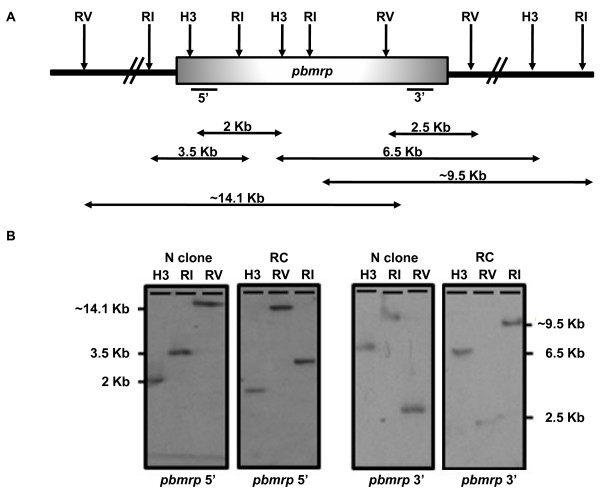
**Southern Blot Analysis of the *pbmrp *Gene**. (A) Diagrammatic representation of the *pbmrp *locus organization. Black arrows above the locus represent the restriction sites within the gene or in flanking areas. Below the coding region, the black lines represent the 5' and 3' specific probes used. Genomic DNA from N clone and RC line was digested with Hind III (H3), Eco RI (RI), and Eco RV (RV). The predicted bands and their sizes are depicted by black arrows. (B) Both probes hybridize to a single band for each of the enzyme used: 14.1 kb for RV, 3.5 kb for RI, and 2.0 kb for H3 (left panel) and 9.5 kb with RI, 6.5 kb with H3, and approximately 2.5 kb with RV (right panel), respectively.

To explore whether *pbmrp *genetic rearrangements and/or an increase in gene copy number contribute to drug resistance in resistant *P. berghei *lines, Southern blot analysis were performed on restricted gDNA from each of the *P. berghei *lines (Figure 5). The 3' probe hybridized to a single band for each enzyme used. Similarly, the hybridization with the 5' probe also resulted in a single band for each enzyme. Results show no difference in the band patterns and hence there is no difference in the genetic organization of *pbmrp *between the drug sensitive N clone and the CQ resistant RC line. In addition, there is no difference in hybridization intensities between the drug sensitive (N clone) and resistant line (RC) indicating the same *pbmrp *gene copy number in both parasite lines.

### Chromosomal location of *pbmrp *in drug sensitive and resistant lines

**Figure 6:**
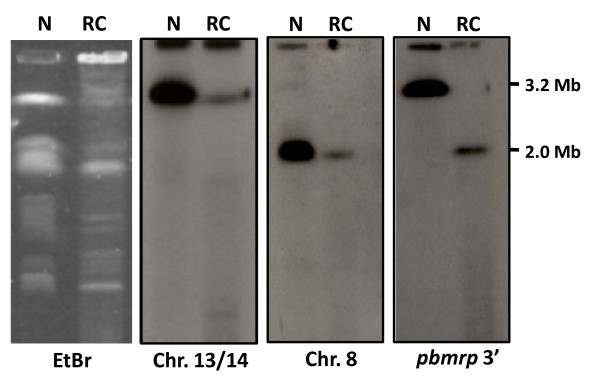
**Chromosomal location of the *pbmrp *gene**. Representative results for the chromosomal location of *pbmrp *gene in the *P. berghei *N clone and RC line. *P. berghei *chromosomes were visualized with ethidium bromide (EtBr) prior to transfer. Chromosome membranes were hybridized with a 3' specific *pbmrp *probe, a chromosome 13/14 specific marker [52] or to a chromosome 8 probe [54]. Note, that the *pbmrp *gene was localized to chromosome 13/14 in N clone and to chromosome 8 in the CQ selected line (RC).

In this study, *pbmrp *was mapped in drug sensitive and drug resistant line. In the drug sensitive, N clone, the *pbmrp *gene was localized to the largest chromosome band corresponding to chromosome 13/14, which co-migrate in *P. berghei *(Figure 6). However, in CQ selected line (RC), the *pbmrp *gene was detected only in chromosome 8 indicating that a translocation event took place. This translocation was observed using *pbmrp *specific probes targetting the 3' terminus.

### Comparison of *pbmrp *expression profiles in drug sensitive and resistant lines

**Figure 7:**
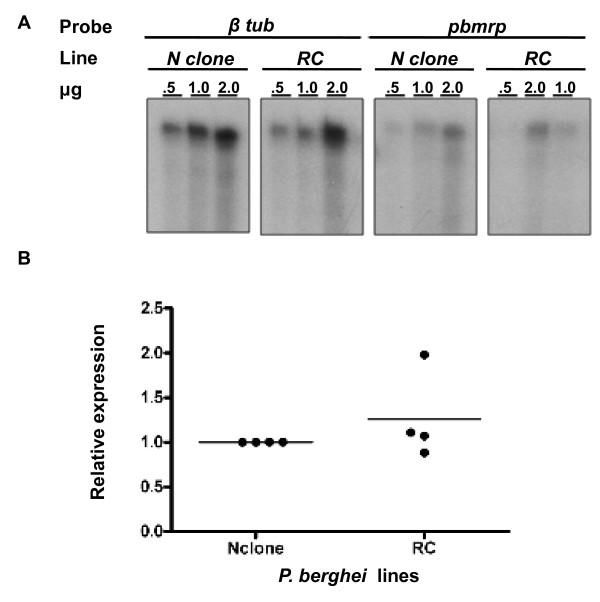
**Determination of *pbmrp *transcript levels in sensitive and drug resistant *P. berghei *parasites**. (A) Representative results of an RPA analysis using total RNA dilutions from the drug sensitive N clone or the CQ selected RC line showing protected fragments with the *βtub *or *pbmrp *riboprobes. *βtub *was included as a normalizing control. (B) Densitometric analysis of *pbmrp *expression. Densities of the normalized *pbmrp *signals were subsequently normalized to the drug sensitive N-clone to estimate the relative mRNA expression levels in RC. The horizontal line represents mean relative expression of four experiments.

RNase protection assays were employed to measure *pbmrp *expression at the level of transcription in the drug sensitive N clone and the drug resistant line, RC. Expression in RC was 1.26 ± 0.49 (mean ± std.dev) relative to N clone *pbmrp *RNA expression levels (Figure 7). Similar *pbmrp *transcription levels were detected in RC when compared to N clone.

## Discussion

Energy-dependent transporters are responsible for maintaining the metabolic homeostasis in organisms. Understanding transporters involved in cellular detoxification and/or drug efflux in *Plasmodium spp. *can provide critical information about their function and their potential as new drug targets. *Plasmodium berghei*, a rodent malaria model, is a proven and valuable tool for studying *Plasmodium *biology as well as the development of resistance to anti-malarials which continues to be global health problem. In this study, a multidrug resistance associated protein gene in *P. berghei *was sequenced. This gene, *pbmrp*, was classified and characterized using bioinformatics. The *pbmrp *gene organization, copy number, and gene expression levels were compared between the drug sensitive N clone and the CQ selected (RC) *P. berghei *line.

The *pbmrp *gene has an intronless ORF of 5820 nucleotides and is predicted to encode a protein comprised of 1939 amino acids. NCBI's Conserved Domain Database classified pbMRP as a member of the ABCC transporter sub-family (Figure 1a). Using the predicted pbMRP sequence, six homologues were identified in four *Plasmodium *species (*P. falciparum, P. vivax, P. knowlesi *and *P. y. yoelii*) by sequence similarity searches in the PlasmoDB and in NCBI. We note that at the time the analyses were performed the annotation of the proteins at NCBI and PlasmoDB is only complete for the pfMRP2 and pfMRP1. For pvMRP1 and pvMRP2 the annotations are ABC transporter and multidrug-resistance associated protein, respectively. The classification as MRP1 and MRP2 is based on the results presented here from similarity searches and phylogenetic analyses. For pkMRP2, pyMRP2 and pbMRP2 the annotations are transporter/putative mrp, ABC transporter, and multidrug-resistance associated protein respectively. As before, classification as MRP2 is based on the results presented here from similarity searches and phylogenetic analyses. At this time, the presence of two *mrp *genes appears to be unique for the two human malaria species, *P. falciparum *and *P. vivax *(Table 2). An additional *mrp *gene may confer a biological advantage for *P. falciparum *or *P. vivax *with respect to fitness or the evolution of drug resistance but this has yet to be proven experimentally. BLAST results support that from an evolutionary standpoint pbMRP is more closely related to pyMRP, pkMRP, pfMRP2 and pvMRP2 rather than to pfMRP1 and pvMRP1 (Table 1). Interestingly, sequence similarity searches against the human genome show that pbMRP is more closely related to the MRP2. Taken together, the fact that pbMRP is more similar to pvMRP2 and pfMRP2 in addition to having the closest relatedness to the human MRP2 confirms that the *P. berghei *transporter is a member of the ABCC MRP2 sub-family. The phylogenetic analysis presented in Table 1 and Figure 4 supports the hypothesis that the MRP2 family is the ancestral family of MRPs in *Plasmodium*. It is noteworthy that at this time the MRP1 sub-family only appears in parasites that infect humans. Given the lack of knowledge about the function of the MRPs in *Plasmodium*, it is not possible to assess the significance of the finding at this time. It is also important to note that the MRP2 clade groups the gene from *P. knowlesi *with that of *P. vivax*, in agreement with published phylogenies [[55] and references therein]. Human MRP2 is involved in the terminal phase of detoxification and excretion of endogenous and xenobiotic organic anions in a unidirectional manner. This protein actively transports glutathione, glucuronate, or sulphate conjugates in addition to a variety of non-conjugated anionic substances including glutathione and glutathione disulfide [56]. Although MRP2 has broad substrate specificity, it has highest affinity for gluconarate and GSH conjugates of lipophilic substances [57,58]. Based on the sequence similarity to MRP2 and taking into account the biological function of this membrane transporter, it would be reasonable to suggest that the biological function of pbMRP might be related to cell detoxification and the secretion of conjugated and/or non conjugated endogenous and xenobiotic anions. In addition, human MRP2 has been shown to confer drug resistance to multiple chemotherapeutic agents in cell lines expressing the recombinant protein or antisense constructs [59-61]. Given that MRP2 confers resistance in human cells in addition to the fact that the overexpression of homologues in *Leishmania *and *T. brucei *[24,26,29] is associated with drug resistance raises the possibility that *pbmrp *may also contribute to this phenomenon.

The pbMRP displays the typical core ABCC/MRP domain organization of two units of a TMD and a NBD (Figure 1c). All human MRPs, the best and most completely characterized members of this sub-family, possess this typical core structure with the exception of MRP1, MRP2, MRP3, MRP6 and MRP7 which have an additional N-terminal region composed of a third transmembrane domain (TMD0) [62,17,64]. The pbMRP does not possess this additional TMD0 (Figure 1b) similar to the other members of human MRP family such as MRP4, MRP5, MRP8 and MRP9 [63,65-67].

The NBD size and organization in the MRPs of the *Plasmodium spp. *studied were similar to those described in other organisms. A high level of similarity was observed in multiple sequence alignments, in particular within the NBDs with the exception of a large, non conserved insertion of approximately 103 amino acids in NBD1 (from position 961 to 1110 in the multiple sequence alignment) (Figure 3, Additional file 3). Characteristic NBD motifs such as the Walker A, Q loop, ABC motif, Walker B, D loop and the H loop were found within the NBDs (Figure 3). All of the characteristic motifs were identified with the exception of the H loop in NBD1. This NBD is unique in the sense that there is an insertion between the ABC motif and the Walker B motif. This insertion was present in all the *Plasmodium *MRP sequences studied. The Walker B motif and the D loop were identified after this large insertion in the multiple sequence alignment. The location and sequence of the Walker B motif in the region following the insertion is similar to that described as the Walker B in pfMRP1 [30]. The D loop could not be identified in the MRP NBD2 in all the *Plasmodium *species studied (Figures 2 and 3). The failure to identify the H loop in NBD1 and the D loop in NBD2 may be due to a lack of conservation in the sequences encompassing these motifs in *Plasmodium*. Both the H loop and the D loop have structural functions within the NBD although their precise role in the molecular mechanism is not clear. The H-loop histidine contacts the bound nucleotide and the D loop may form a hydrogen bond with the Walker A backbone [13,68]. Remarkably, the sequences characteristic of an ABC transporter NBD were highly conserved among the *Plasmodium *species studied but these are not strictly conserved when compared to the consensus sequences described for these motifs in vertebrates (Table 2).

The structure and function of MRP2 genes in *Plasmodium *has not been described. In neoplastic cells as well as in *Leishmania*, gene duplication and/or overexpression correlate with the drug resistant phenotype [69,70]. In this study, the *pbmrp *gene was shown to have has similar gene organization and the same gene copy number in both the drug sensitive N clone and the CQ selected RC line (Figure 5). In addition, this gene was not differentially expressed at an mRNA level between these two lines (Figure 7). However, amplification or increased expression might have been lost upon removal of drug pressure given that RC line was not under continuous CQ pressure in these experiments. In primary cultures of rat brain endothelial cells treated with dexamethasone, increased expression of *mrp2 *at the mRNA and protein level was detected in a concentration dependent manner. This increase in expression was reversible in the absence of drug pressure [71]. In a CQ selected *P. falciparum *clone, an increase in the size of chromosome 3 was observed with increased CQ pressure as a result of DNA amplification whereas removal of CQ selection resulted in the return of chromosome 3 to its original size [72]. Single nucleotide polymorphisms within *pfmrp1 *have been associated with anti-malarial resistance in field isolates [5,6,32]. Nevertheless, mutations within *pbmrp *may also contribute to the CQ drug resistance phenotype.

The *pbmrp *locus was mapped to chromosome 13/14 in the drug sensitive N clone. Similarly, synteny analysis between the *P. falciparum *and the *P. y. yoelii *genome indicate that *pymrp *is located in chromosome 14 [73]. However, a striking finding in our study was that unlike N clone, the CQ selected line RC exhibited a chromosomal translocation of this gene to chromosome 8 (Figure 6). In a CQ resistant *P. falciparum *isolates subjected to mefloquine selection and constant drug pressure, *pfmdr1*, a member of the ABCB transporter sub-family, was amplified resulting in an increase in size of chromosome 5 [74]. A partial chromosomal duplication of the region containing the *mdr1 *gene was described in a *Plasmodium chabaudi *line selected for a stable mefloquine-resistance, where a second copy was translocated from chromosome 12 to chromosome 4 [75]. Pyrimethamine selection of a *P. chabaudi *line resulted in the duplication of the DHFR gene and a rearrangement of chromosome 7 [76]. The detection of *pbmrp *solely in chromosome 8 in the RC line and the fact that this chromosome appears to be of similar size as its counterpart in the drug sensitive N clone support that our observation is a translocation event without gene duplication. Previous work mapped the *pbggcs *gene to chromosome 8 in the *P. berghei *lines studied [54]. Co-transfection experiments in *Leishmania *with the genes for PGPA and GGCS demonstrated that they work synergistically to confer resistance to antimony [77].

Therefore, the relocation of the *pbmrp *gene to the same chromosome of a potential linked gene, *pbggcs*, is intriguing and deserves further examination. It remains to be established whether pbMRP transports anions such as GSH, and/or GSH S-conjugates and whether it confers resistance to any anti-malarials.

## Conclusion

The *P. berghei mrp *gene was sequenced and compared to homologues of this gene in other *Plasmodium *species and organisms. Using bioinformatics the *pbmrp *gene was found to have the highly conserved ABC motifs and was classified as an ABCC/MRP2 type membrane transporter.

There were no differences in gene organization, copy number, or level of expression when comparing a drug sensitive and a CQ selected drug resistant *P. berghei *line. A chromosomal translocation of the *pbmrp *gene from chromosome 13/14 to chromosome 8 was observed when comparing the drug sensitive N clone and the CQ resistant line RC.

The fact that some of these proteins are involved in the drug resistance phenomenon in other species justifies further investigations on the potential contribution of ABC transporters to anti-malarial resistance in *Plasmodium*. The results of this study justify the examination of the role of the *Plasmodium berghei *multidrug resistance associated protein in *Plasmodium *detoxification pathways. Future research on membrane transport mechanisms and intracellular biochemical processes mediated by these transporters could result in the identification of novel drug targets or multidrug treatment strategies to combat malaria.

## Competing interests

The authors declare that they have no competing interests.

## Authors' contributions

MGP participated in the work for the biological assays, performed the bioinformatic analyses, and drafted the manuscript and figures. ACS contributed to the study design, participated in the work for the biological assays, performed the sequencing experiments, carried out some bioinformatic analyses, and helped draft the manuscript. ACS submitted part of this work in partial fulfillment of her doctoral dissertation. RGM designed, supervised, and carried out part of the bioinformatics analyses. RGM carried out the phylogenetic analysis. RGM also helped draft the manuscript and figures. AES contributed to the design of the study, was responsible for the supervision of the work, and helped draft the manuscript. All authors read and approved the final manuscript

## Supplementary Material

Additional file 1 **Primers used in *pbmrp *sequencing reactions**. Table that includes all the primer combinations used to sequence the *pbmrp *gene in the *Plasmodium berghei *N clone line. Forward and reverse primers are prefaced with an F or an R, respectively. Click here for file

Additional file 2 **Complete *pbmrp *nucleotide sequence and predicted amino acid sequence**. The complete nucleotide sequence of the *pbmrp *gene from the *Plasmodium berghei *line is presented together with the predicted protein sequence. Click here for file

Additional file 3 **Complete multiple sequence alignment the MRP sequences from five *Plasmodium *species**. The complete multiple sequence alignment for the MRPs obtained from five *Plasmodium *species: *P. y. yoelii, P. berghei, P. vivax, P. knowlesi, and P. falciparum*, is show. Click here for file
