# Supplementary material for: Identification and bioinformatic characterization of a multidrug resistance associated protein (ABCC) gene in Plasmodium berghei
Source: Malar J. 2009 Jan 2;8:1. doi: 10.1186/1475-2875-8-1 (PMC2630995; doi:10.1186/1475-2875-8-1)
Supplement: Additional file 1 — Primers used in pbmrp sequencing reactions. Table that includes all the primer combinations used to sequence the pbmrp gene in the Plasmodium berghei N clone line. Forward and reverse primers are prefaced with an F or an R, respectively. [file 1475-2875-8-1-S1.pdf]

Primers	Sequence (5'-3')
F-pbmrp1<br>R-pbmrp1	ATAAATAGATGAACCGAAAAATG<br>AAAGTGCATAACAGTTACTTCC
F-pbmrp2<br>R-pbmrp1	TAATATAGATAAAAATGAGGGGG<br>AAAGTGCATAACAGTTACTTCC
F-pbmrp3<br>R-pbmrp2	GCCTATACCTTCATACAACCAGT<br>ACATTCCATTAGCCATTATTGCAG
F-pbmrp4<br>R-pbmrp3	TGAAACAAGCCAAAGTAATGCC<br>CACACGGGCATTACTTATAGGTCC
F-pbmrp5<br>R-pbmrp4	TATGTGAATTCATATCAAGTATG<br>ACTGGTTTAAAATTACAATTTTC
F-pbmrp6<br>R-pbmrp4	TAGAAGGACCTATAAGTAATGC<br>ACTGGTTTAAAATTACAATTTTC
F-pbmrp7<br>R-pbmrp5	AAAATAGTAAAATGAACGAAAT<br>AGATTCTGAAAAACGTACTGATTTC
F-pbmrp8<br>R-pbmrp6	TTGATAATATGAAATCAGTACG<br>GATTATTTTGATGACATAAT
F-pbmrp9<br>R-pbmrp7	GATCAAGTATTTTATATGTCAATGTCAT<br>GGAGAATGAGAACATAAATATCCTC
F-pbmrp10<br>R-pbmrp8	TTTTAAAAAATATTCAGTAGCA<br>CATCAATAATTTCATTATCAGA
F-pbmrp10<br>R-pbmrp9	TACAATAGTTATGGCAATATTAG<br>CTACTGAATATTTTTTAAAACA
F-pbmrp11<br>R-pbmrp8	CTAATGTCTGATGAT<br>CATCAATAATTTCATTATCAGA
F-pbmrp12<br>R-pbmrp10	CTAATGTCTGATGAT<br>CGCTTTATACGGTTTGTATGCA
