# Supplementary material for: Identification and bioinformatic characterization of a multidrug resistance associated protein (ABCC) gene in Plasmodium berghei
Source: Malar J. 2009 Jan 2;8:1. doi: 10.1186/1475-2875-8-1 (PMC2630995; doi:10.1186/1475-2875-8-1)
Supplement: Additional file 2 — Complete pbmrp nucleotide sequence and predicted amino acid sequence. The complete nucleotide sequence of the pbmrp gene from the Plasmodium berghei line is presented together with the predicted protein sequence. [file 1475-2875-8-1-S2.pdf]

```
  1 - ATG AAC CGA AAA ATG AAG AAA AAT TAT ATA GAT AAG AAT AAT GAG GGG GGA AAG GAG ATG  - 60
  1 -  M   N   R   K   M   K   K   N   Y   I   D   K   N   N   E   G   G   K   E   M   - 20

 61 - CCT AAG AAT AAA TTA TCA TTT GTT AAA TTT ATT ACT TTT CAT TGG ATA ACC AAA TTA ATA  - 120
 21 -  P   K   N   K   L   S   F   V   K   F   I   T   F   H   W   I   T   K   L   I   - 40

121 - AAT AGT ATA AAT AAT GCT GAA GAT TTT ATT TTG CCA AAT ATT GGA AGG AAA CCA ATC ATA  - 180
 41 -  N   S   I   N   N   A   E   D   F   I   L   P   N   I   G   R   K   P   I   I   - 60

181 - GGG TAT TAT GAA TAT TAT TTG ATG AAA AAT TTA AAA GTT TTC AGA AAA AAA AAA AAA TCG  - 240
 61 -  G   Y   Y   E   Y   Y   L   M   K   N   L   K   V   F   R   K   K   K   K   S   - 80

241 - TTT ATA AGT CGA TTT TTT TCC AAA ATT TTG AGT TTT ACT GTT AAT TTA AAA AAA AGT GAT  - 300
 81 -  F   I   S   R   F   F   S   K   I   L   S   F   T   V   N   L   K   K   S   D   - 100

301 - AGA AAT AAA AAA AAT AGG AAT AGA ATT AAA GAT GAT GAT TAT TTT TAT GAA TAT AAC AGA  - 360
101 -  R   N   K   K   N   R   N   R   I   K   D   D   D   Y   F   Y   E   Y   N   R   - 120

361 - GGA ATT ATT GCG GCA TTG ACT TAT ACT TTC AAA CAA CCA GTG TTG ATT ATA TCA CTC CTT  - 420
121 -  G   I   I   A   A   L   T   Y   T   F   K   Q   P   V   L   I   I   S   L   L   - 140

421 - TAT ATA TTA CAC GCA TTA TTT TTA GTG TTT GTT GCT ATT TGT ATA GAA AAA TAT ATA TCA  - 480
141 -  Y   I   L   H   A   L   F   L   V   F   V   A   I   C   I   E   K   Y   I   S   - 160

481 - ATC ATA AAA GGA CAT CAC GTT TTT TCT CCA TTA TTA CAA TCT AAA ACT GCA AAA CTA TTA  - 540
161 -  I   I   K   G   H   H   V   F   S   P   L   L   Q   S   K   T   A   K   L   L   - 180

541 - TCT GCA TTT GTT CTT ATA ATG GTA TTA TCC TTG AAT TTA TTT ATG GAT TCG GCC GTT AGT  - 600
181 -  S   A   F   V   L   I   M   V   L   S   L   N   L   F   M   D   S   A   V   S   - 200

601 - TAT ATA CAT AAC AAA CTG ATA ATT GAT ATG GAA GTA ACT GTT ATG CAC TTT TTG TAT AAA  - 660
201 -  Y   I   H   N   K   L   I   I   D   M   E   V   T   V   M   H   F   L   Y   K   - 220

661 - ATA AAT ATG GGA GTA TTT AAT CAT TGT ATA TTA AAC CAA TAT CAT GAT GAT AAT AAT AAT  - 720
221 -  I   N   M   G   V   F   N   H   C   I   L   N   Q   Y   H   D   D   N   N   N   - 240

721 - AAT TTA CAT GAA ATA AGT AAC AAT AAT AGT GAA AAT AGT ACT TGC GCA AAT ATA GAT TCT  - 780
241 -  N   L   H   E   I   S   N   N   N   S   E   N   S   T   C   A   N   I   D   S   - 260

781 - TAT AAA AAT AAT ACA CAT GGC ACA TCC AAA AAT TAT ATT ATA AAA GTT GAA AAT GAA GAT  - 840
261 -  Y   K   N   N   T   H   G   T   S   K   N   Y   I   I   K   V   E   N   E   D   - 280
```

841 - GAA AAT GCA GAA TAT AAA AAT GAA ACA AAT CAA AGT AAT GCC AAT ACC ACT ATA AGC ACA - 900
281 - E N A E Y K N E T N Q S N A N T T I S T - 300

901 - AAT AAC CTT ACC GAT TTT TCA AAA AAA AAT GAT TTA AAT AAT AAA TTA GAA GAT AAT GAA - 960
301 - N N L T D F S K K N D L N N K L E D N E - 320

961 - AAT GTG ATT GAA CAT AAT AAT GGC GAT GGT GGT ATT AAC ATA TAT AAT ATT ATG TTT ATA - 1020
321 - N V I E H N N G D G G I N I Y N I M F I - 340

1021 - GAT ACA CCA TCT TTA ATA TAT TTT ATA TCT TCT GCA ATA ATA GCT AAT GGA ATG CTT ATT - 1080
341 - D T P S L I Y F I S S A I I A N G M L I - 360

1081 - AAA TTG GTA ATA TCA TTT TAT ATG TTT TAT CAT AAA ATG GGA AAA GAT TCT ATT ATA ATT - 1140
361 - K L V I S F Y M F Y H K M G K D S I I I - 380

1141 - GGA ATA TTT TTA GTA TTA TTA TTG TAT GGA ATT ATA TTA TTA TGC GAA TTA ATA TCA AGT - 1200
381 - G I F L V L L L Y G I I L L C E L I S S - 400

1201 - ATG CTT AAA AAA AAA TAC TTA AAA TAT CAG GAT AAA AGA ATA GAT AAC ATG AAT CAT GTT - 1260
401 - M L K K K Y L K Y Q D K R I D N M N H V - 420

1261 - TTA AAA GAA TAT AAA TTA ATG AAA ATG TTT AAT TGG GAA TCT ATA GCA TTT GAT TAT GTA - 1320
421 - L K E Y K L M K M F N W E S I A F D Y V - 440

1321 - AAT AAA TTT AGG GAA AAG GAA TTG AAG TAT TGT AAA TAC AGA ATT TAT TTG AGT TCT ATA - 1380
441 - N K F R E K E L K Y C K Y R I Y L S S I - 460

1381 - AGT AAT TAT ATT AAT GCC ATT TCC GTT CAT TGT GTA GAA ATA GTT ATA TTT TTT GTT TAC - 1440
461 - S N Y I N A I S V H C V E I V I F F V Y - 480

1441 - ATT AGA AAC AAG CTA AAT AAT AAT GAT CAA ATT GAT GTT AAT TCG GTA ATT ACA CCC CTT - 1500
481 - I R N K L N N N D Q I D V N S V I T P L - 500

1501 - TTT GTT TAT AAA TCG TTA ATT AAC GGA ATT GTA AGT TTT CCA ACT ATA TTC AAT AAT TTA - 1560
501 - F V Y K S L I N G I V S F P T I F N N L - 520

1561 - TTA GAA GGA CCT ATC AGT AAT GAC CGT GTG AAT AAA TAT ATT AAC CAT TAT TTT CAT GAC - 1620
521 - L E G P I S N D R V N K Y I N H Y F H D - 540

1621 - AAT ATC TCC AGT AAG CTT TTT TAT TCT AAA ATT AAA AAT GAA AAA AAA AAT AGC CAT TTA - 1680

```
541 - N   I   S   S   K   L   F   Y   S   K   I   K   N   E   K   K   N   S   H   L   - 560

1681 - AAA AAA AAA AAT AAT AAT TAT ATG AAT ATG CAA GGT AAA TCA GAG AAA ATT ATT CCT TCC  - 1740
 561 - K   K   K   N   N   N   Y   M   N   M   Q   G   K   S   E   K   I   I   P   S   - 580

1741 - AAA AAA AAT ACA TTT TAT ACT AGC TTT TTA AAC ATG TTT TCA AAT GAC GAT TAT TAT TAT  - 1800
 581 - K   K   N   T   F   Y   T   S   F   L   N   M   F   S   N   D   D   Y   Y   Y   - 600

1801 - AGT AGT AAT TAT AAT GAT CAT GGT AGC AAT ACA ACG AAT TTT GAA TGG GAA AAT GAA AAT  - 1860
 601 - S   S   N   Y   N   D   H   G   S   N   T   T   N   F   E   W   E   N   E   N   - 620

1861 - AAT AAA ATA TAT CGA AAT AAT AGT GGC GAC CAT ATT AAT AAC AAA ATA GTA ACA ATA GAC  - 1920
 621 - N   K   I   Y   R   N   N   S   G   D   H   I   N   N   K   I   V   T   I   D   - 640

1921 - ATT TTA AAT AAA AAT AGT AAA ATG AAC GAA ATA AAT GAG GGT ATT AAA TCA AAA AAT ATT  - 1980
 641 - I   L   N   K   N   S   K   M   N   E   I   N   E   G   I   K   S   K   N   I   - 660

1981 - CTT GAT GAA AAA TAT GGA ATA GAT AAA AAA ACA ATT ATA AAA TTG GAA AAT TGC TAT TAT  - 2040
 661 - L   D   E   K   Y   G   I   D   K   K   T   I   I   K   L   E   N   C   Y   Y   - 680

2041 - AAA CCA GTG AAA TTT GAA AAC AAT TAT AAT CAA TCC AAA AAT ATG AAA TTG AAA AAT GTA  - 2100
 681 - K   P   V   K   F   E   N   N   Y   N   Q   S   K   N   M   K   L   K   N   V   - 700

2101 - AAT TTT ACA TTA AAA AAT AAT ACT ATA GCA ATA ATA ATA GGT GAT ATT GGA TCA GGG GAA  - 2160
 701 - N   F   T   L   K   N   N   T   I   A   I   I   I   G   D   I   G   S   G   E   - 720

2161 - ACA TTA TTT TTT AAT TCC ATT CTT GGG AAG TTT AAA TTA TTA AAT GGA AAT TAT TAT ATT  - 2220
 721 - T   L   F   F   N   S   I   L   G   K   F   K   L   L   N   G   N   Y   Y   I   - 740

2221 - AAA AAT TTT ATA TAT GAT ATG CCA GTT TTA TAT GCA CCT CAA ATT AAT TGG CTA TCT GAT  - 2280
 741 - K   N   F   I   Y   D   M   P   V   L   Y   A   P   Q   I   N   W   L   S   D   - 760

2281 - GGG ACG ATC AGA TCA ATG ATA ACA TTT GAA AAT GAA TTC GAT CCA TAT ATT TAT TAT TTA  - 2340
 761 - G   T   I   R   S   M   I   T   F   E   N   E   F   D   P   Y   I   Y   Y   L   - 780

2341 - GCT ATT TCG CAA AGT GAG CTA ATA AAT GAT ATA TAT TCT TTT AAA AAT TTG GAT ATG CGA  - 2400
 781 - A   I   S   Q   S   E   L   I   N   D   I   Y   S   F   K   N   L   D   M   R   - 800

2401 - TAT GTA AAT GAT GAA CAT AGT TTA AGT AAA GGG CAA AAA TCG AGA ATA TCA TTA GCA CGA  - 2460
 801 - Y   V   N   D   E   H   S   L   S   K   G   Q   K   S   R   I   S   L   A   R   - 820
```

2461 - TCT TTA TAT TAT CAT TAT ATA AAT ATG AAA CAA TTA CGT ACT GAA TAT ATA GAA ATG AAA - 2520
821 - S L Y Y H Y I N M K Q L R T E Y I E M K - 840

2521 - AAT GCT GAA GAA AGC ACA TTT GAT AGT ACA AAG GAA AGT AAT TTT GGC TTA AAT AAA TTC - 2580
841 - N A E E S T F D S T K E S N F G L N K F - 860

2581 - GCA ACA AAT TAT TAT GAT AAA AAT ATT GAT AAT ATG AAA TCA GTA CGT ATT TCA AAA TCC - 2640
861 - A T N Y Y D K N I D N M K S V R I S K S - 880

2641 - ATT AAA AAT TTC ACA TTG CAT AAC GAT AAT CAA TTT GAA AAT TCA ATA AAC CCT AAT ATA - 2700
881 - I K N F T L H N D N Q F E N S I N P N I - 900

2701 - TAT AAT GAT GAG TCT GAA CAA ATT AAA GGA AAT AAT AAA AAT AGT AAA AGC GAT ACT AAC - 2760
901 - Y N D E S E Q I K G N N K N S K S D T N - 920

2761 - AGT TTT ATT AGT GAT TGT AAA TTT TCA TAT CCA GAT TTA CAA TTC TTG ATG AAT AAT AAC - 2820
921 - S F I S D C K F S Y P D L Q F L M N N N - 940

2821 - TAT TTA AAA GAC TGT TTA GAG AAA AAC GAT ATG TCA TAT TTA TAT TTA TTA GAT GAT ATA - 2880
941 - Y L K D C L E K N D M S Y L Y L L D D I - 960

2881 - TTC ACT TCA TTA GAT CCA TAT ATT TCA AGA AAT ATA TTT TAT AAT TTA TTT TGT GAT AAA - 2940
961 - F T S L D P Y I S R N I F Y N L F C D K - 980

2941 - GAA AAA TTG AAA ATT ATT AAA AAC CAT TGT GGA ATT GTC ATA ACA ATC AAT GAA AAT GCA - 3000
981 - E K L K I I K N H C G I V I T I N E N A - 1000

3001 - TTT AAT AGT TTT ATA ATG AAA GAC ATT ATA GAA AAT ATT CAA TAT AAT GTG GAT ATA TAT - 3060
1001 - F N S F I M K D I I E N I Q Y N V D I Y - 1020

3061 - AAA TTG GAA AAT GGA TCA TTA GAC TTT CAA GGT AAT ATA AAT GAA TAT ATA CAA AAG AAA - 3120
1021 - K L E N G S L D F Q G N I N E Y I Q K K - 1040

3121 - AAT ATA CAA ATA AAA GCT TCT GAT GTA ATA ATT AAA AAA GAA ACA GAA AAT GAA AAA AGA - 3180
1041 - N I Q I K A S D V I I K K E T E N E K R - 1060

3181 - GAA AAA ATG CTT TCA TTT TTC AAA AAA ACA GGA ATA TCA ATG AAT TGT TCT AAA ATG AGT - 3240
1061 - E K M L S F F K K T G I S M N C S K M S - 1080

3241 - GAT CAA GTA TTT TAT ATG TCA ATG TCA TTA GAA TTA TGC TAT CAA AAT AAT CCA CAA ACA - 3300
1081 - D Q V F Y M S M S L E L C Y Q N N P Q T - 1100

```
3301 - GAA GTT GAA ATT ACA AAT AGT GAT AAT ATT GAG AGC TCT TTG AGC ACT AGT GTT ATT TAC  - 3360
1101 -  E   V   E   I   T   N   S   D   N   I   E   S   S   L   S   T   S   V   I   Y   - 1120

3361 - AAA AGA GAT AAT TCA TAT TTC GAT AAA AAT AAT ATA ATA TAT AAT AAA ATT ATT TTA TTG  - 3420
1121 -  K   R   D   N   S   Y   F   D   K   N   N   I   I   Y   N   K   I   I   L   L   - 1140

3421 - AAG GAA TTG AAA CTT TTA CAC CAT ATT AAA GCA GAA GCT AAT AAT GAA ACA CCC AAT ATA  - 3480
1141 -  K   E   L   K   L   L   H   H   I   K   A   E   A   N   N   E   T   P   N   I   - 1160

3481 - AAT AAA AAA TTT ACT ATA CTA ATG AAT AAT CAT TTT AAT AAA ATT GTA AAT GAA TCA ATT  - 3540
1161 -  N   K   K   F   T   I   L   M   N   N   H   F   N   K   I   V   N   E   S   I   - 1180

3541 - TTA AAG GAT CGA ATT CAT AAT ATT AGT ACA TAT GAA ATG GCA AAA ATA AAG AAA GAT CAA  - 3600
1181 -  L   K   D   R   I   H   N   I   S   T   Y   E   M   A   K   I   K   K   D   Q   - 1200

3601 - ATT AGG AAA TAT ATT GGA AAT TTT ATT GTA GAT ACA AAA GGA AAT AAT GAA ATG AAA TGT  - 3660
1201 -  I   R   K   Y   I   G   N   F   I   V   D   T   K   G   N   N   E   M   K   C   - 1220

3661 - TTA AAA AAC AAA AAA ACA CAT ATA AAC GAA TTT GAA GAA ATT AAT GCA ATG TTA AAA AAA  - 3720
1221 -  L   K   N   K   K   T   H   I   N   E   F   E   E   I   N   A   M   L   K   K   - 1240

3721 - AAC TTA AAA GAA CAT TAT ACG TAT TAT AAT AAT TAT ATC AGA TAT AAT AGT AAT AAT GAT  - 3780
1241 -  N   L   K   E   H   Y   T   Y   Y   N   N   Y   I   R   Y   N   S   N   N   D   - 1260

3781 - ACT AAT ATA GAT GTA GCT AGG AAA GGA AAT ATA AAT TTT GAG ACA TTT AGA TGG TAT TTT  - 3840
1261 -  T   N   I   D   V   A   R   K   G   N   I   N   F   E   T   F   R   W   Y   F   - 1280

3841 - AGA AGT ATT GGT AAT GCA ATT ATA ATT TGT ATT ATT ATA TTT ATC ATA TTT TCT ATA TTT  - 3900
1281 -  R   S   I   G   N   A   I   I   I   C   I   I   I   F   I   I   F   S   I   F   - 1300

3901 - TTA GAT GAA GTA AAA AAT ATG TTA TTA TTT TTA GTT AGC GCG TTA TTA AAA ACA AAA GAC  - 3960
1301 -  L   D   E   V   K   N   M   L   L   F   L   V   S   A   L   L   K   T   K   D   - 1320

3961 - AAA TCT TAT GAA GAA ATA ATA CAA ACA AAA TTA GTA TAT TTA AAA TAT TTT ATT TTA TTG  - 4020
1321 -  K   S   Y   E   E   I   I   Q   T   K   L   V   Y   L   K   Y   F   I   L   L   - 1340

4021 - CCA GCA TTA TCT TTA GTA ACC ACT TTT ATA TCT TAT ATG GTA ATT GCA CAT GGA ATA ATG  - 4080
1341 -  P   A   L   S   L   V   T   T   F   I   S   Y   M   V   I   A   H   G   I   M   - 1360

4081 - ATA TCA GCA AAG GCA ATT CAC ACA GAA GTA TTT AAA AGT ATG CTA TAT GCC CCT ATT CCT  - 4140
```

1361 - I S A K A I H T E V F K S M L Y A P I P - 1380

4141 - GCA TTT TAT AGT CAT AAT ATA GGG AAT ATA ATT AAT AGA TTT ATA ATA GAT ATT CAC ACA - 4200
1381 - A F Y S H N I G N I I N R F I I D I H T - 1400

4201 - TTA GAT AAT GGG ATA ATT AAA CGA TGT TAT AAA TCA TTT TTT ACT GTT TCC AAA TTT ATA - 4260
1401 - L D N G I I K R C Y K S F F T V S K F I - 1420

4261 - TCA ACC GTT ATT TTA TTA ATA TTT ATG TTT AAG AAA ACA TAT ATT ATG TTA CCA TTT ATT - 4320
1421 - S T V I L L I F M F K K T Y I M L P F I - 1440

4321 - ATA TTA ATT GTT TAT TAT GGA ATT TTC AAA AAA TAT TCA TTA GCC TGT AAA GAA GCT CAA - 4380
1441 - I L I V Y Y G I F K K Y S L A C K E A Q - 1460

4381 - AGA GGA TAT TTA TGT TCG CAT TCT CCA ATT TGC TCC ATA TTT AGT AAT ACT ATA CAT GGA - 4440
1461 - R G Y L C S H S P I C S I F S N T I H G - 1480

4441 - AAA GAT ATT ATA AAT TTA TAT AAA AAA AAT TAT TGT ATT TTG AAA AAA TTT GAA AAC AGT - 4500
1481 - K D I I N L Y K K N Y C I L K K F E N S - 1500

4501 - ATT TAT GCA CTT CGA AAT TTT ACT TTA TTC AAA TGG GGT ATA ACT GCA TGG GCA TCG TTA - 4560
1501 - I Y A L R N F T L F K W G I T A W A S L - 1520

4561 - TAT ATA CAA TTA GTA AGT TTA TGC TTA ACA TCA TTT TAT ATT TTA TAT CCG CAT GTT TTT - 4620
1521 - Y I Q L V S L C L T S F Y I L Y P H V F - 1540

4621 - TCA ATA TTC AAA AAT TCT TCT GAG CAT ATT GAT ATA GAT GTA GAT GAT TAT ATA AGT TAT - 4680
1541 - S I F K N S S E H I D I D V D D Y I S Y - 1560

4681 - AGT GAA GTT ATT GGA TAT TGT ATA ACA TTT TCA TGT AGT TTA GGA TAT ATA ATA AAA TCA - 4740
1561 - S E V I G Y C I T F S C S L G Y I I K S - 1580

4741 - TTT TTA TAT GAT TAT ACA CAT GTA GAA AAA GAA ATG TGC AGT ATT CAG AGT TTA CAA GAA - 4800
1581 - F L Y D Y T H V E K E M C S I Q S L Q E - 1600

4801 - TTA TCA AAA ATA AAA AAT ATT AGT GAT GAA ACA AGT TTT ACT CAA ATT GAA AAT CAT GAA - 4860
1601 - L S K I K N I S D E T S F T Q I E N H E - 1620

4861 - TAT TCA TAT GAG AAT AAT AAA AAC GTT GAA AAT GCT AAT TAT CTA AAT AAC AAA ACT GAG - 4920
1621 - Y S Y E N N K N V E N A N Y L N N K T E - 1640

4921 - GAT GGC ATA TTA ATA ACT CCA AGC CAA TTT CCA AAA TCA AAA TAT GGG CTT GAA TTT AAA - 4980
1641 - D G I L I T P S Q F P K S K Y G L E F K - 1660

4981 - AAT GTT TAT GTT AGT TAT AAA AAA AAA GTA TAT ATA GAT AAA TTA AAA AAT ATA TAT TAT - 5040
1661 - N V Y V S Y K K K V Y I D K L K N I Y Y - 1680

5041 - TAT GCA AAT GAA AAA TCA TGT TTA AGG AAT ATA AAT TTT TAT GCT TTA AAA AGT CAA AAC - 5100
1681 - Y A N E K S C L R N I N F Y A L K S Q N - 1700

5101 - ATT GGA ATT ATT GGA AAA TCT GGA GCA GGA AAA AGT ACA ATA GTT ATG GCA ATA TTA GGA - 5160
1701 - I G I I G K S G A G K S T I V M A I L G - 1720

5161 - TTA ATA TCT ACA TCA AAA GGG GAA ATA AAA ATT GAT GGA AGG GAT ATA AGA AGT ATT CCC - 5220
1721 - L I S T S K G E I K I D G R D I R S I P - 1740

5221 - TTA AAT GAA AAA AAA AAA ATA ATA GGT ATT TTA CCT CAA TCA TCT TTT GTT TTT TCA CAT - 5280
1741 - L N E K K K I I G I L P Q S S F V F S H - 1760

5281 - TGG AAT ATA AGA ACT TAT ATA GAT CCT TAT CAA AAA TTT TCT GAT AAT GAA ATT ATT GAT - 5340
1761 - W N I R T Y I D P Y Q K F S D N E I I D - 1780

5341 - GCT TTT GAA ACA ATT GGA ATA AAC CTT ACG TGT GCA GAT TTA AAT AAA TAT ATA TAC AAA - 5400
1781 - A F E T I G I N L T C A D L N K Y I Y K - 1800

5401 - ACA AAA AAA AAA ATG AAT GAA TAT GAT AAA TCT AAA TAT ACA AAA ACA AAT AAA AAA TAT - 5460
1801 - T K K K M N E Y D K S K Y T K T N K K Y - 1820

5461 - AAT GAA AAT TAT ATA TTA ATG TCC GAT GAT TCA ATA CGA TAT TTA TCA TTA GTA CGA ATA - 5520
1821 - N E N Y I L M S D D S I R Y L S L V R I - 1840

5521 - TAT TTG AAC AGA AAT AAT TAT AAG TTA TTA TTG ATT GAT GAA ATA CCT GTT GTG AAT TTT - 5580
1841 - Y L N R N N Y K L L L I D E I P V V N F - 1860

5581 - AAT AAA AAT AAT AGC GAA TTT AAT AAT TTT TTT ACA AAA AAT TTA AAG CCA TTT GAT TAT - 5640
1861 - N K N N S E F N N F F T K N L K P F D Y - 1880

5641 - ATA ATT GAA AAT TAT TTT AAA CAT ATT ACT ATT TTA ATC ATT TCC CAT GAT ACA AGA ACT - 5700
1881 - I I E N Y F K H I T I L I I S H D T R T - 1900

5701 - CTA TCA TCT TGT GAT TTT ATT TGT GTT GTT TCT AAG GGG GAG ATT GTA TAT AAA TGT AAT - 5760
1901 - L S S C D F I C V V S K G E I V Y K C N - 1920

5761 - TAT TCA GAC GTA GAA ACA CAA ACA CAG CTA GCT AAT ATT ATA CAG GAC CAG GCA AAT TGA - 5820
1921 - Y S D V E T Q T Q L A N I I Q D Q A N * - 1940

5821 - CGT TTA AGA ATG ATA TTC ATA AAA AAA AAT ATA TAT ATC AAT TTA CTA TTT TTA TTA ATT - 5880
1941 - R L R M I F I K K N I Y I N L L F L L I - 1960

5881 - AAA TTA TCA TGA ATT ATA CAT TTT TTA ATT GAA ACA AAA GAG GAG GCA CTT TTT AAT CAA - 5940
1961 - K L S * I I H F L I E T K E E A L F N Q - 1980

5941 - TTT TAA GAT TAT TAA CGT GAT TAA ATA TAT AAT ATT TTT TTT CTC TTT TAT TAA TAT TTT - 6000
1981 - F * D Y * R D * I Y N I F F L F Y * Y F - 2000

6001 - AAA TAA CAA ATA CGG AGT TAA AAT GTA CGA AAA AAT TTT TAA AAG TTT ATT TAA TTG TAA - 6060
2001 - K * Q I R S * N V R K N F * K F I * L * - 2020

6061 - AGC AAC TGA ATG TAT AAT AAA TTT GTG CAT ATA TAT ATA TAT ATA TGT GTA TAT TTT ATT - 6120
2021 - S N * M Y N K F V H I Y I Y I C V Y F I - 2040

6121 - TTT AAT TAA ATA TAA AAG TAT GAA AAG AAA AAA GCA TAT GTA TTA TAT GCA TAC AAA CCG - 6180
2041 - F N * I * K Y E K K K A Y V L Y A Y K P - 2060

6181 - TAT AAA GCG TAA TA - 6194
2061 - Y K A * X - 2080
