# Supplementary material for: Identification and bioinformatic characterization of a multidrug resistance associated protein (ABCC) gene in Plasmodium berghei
Source: Malar J. 2009 Jan 2;8:1. doi: 10.1186/1475-2875-8-1 (PMC2630995; doi:10.1186/1475-2875-8-1)
Supplement: Additional file 3 — Complete multiple sequence alignment the MRP sequences from five Plasmodium species. The complete multiple sequence alignment for the MRPs obtained from five Plasmodium species: P. y. yoelii, P. berghei, P. vivax, P. knowlesi, and P. falciparum, is show. [file 1475-2875-8-1-S3.pdf]

```
                  *        20         *        40         *        60         *        80
pbMRP  : ------------MNRKMKKNYIDKNNEGGKEMPKNKLSFVKFITFHWITKLINSINNAEDFILPNIGRKPIIGYYEYYLM :   68
pyMRP  : -----------------MKKNNIDKNEGEKEVDKNKLSFINFFTFHWITKLINSINKTEEFVLPNIGRKPIIGYYEYYLM :   63
pkMRP  : ------------MNGKNAKGKDGARKQGKQGSLIASMSWFGFITFEWITIILNKLKAEDNFTLPKVEEDAAIEYYAYNLG :   68
pvMRP2 : MACNVVAPPPGTMSGRTAKGKGAARQRG---SVIANISWFGFITFEWITIILNKLRREDNFSLPKIEEDAAIEYYTCKLG :   77
pfMRP2 : ---------------MMRRRSVYNFDQGKHGYLMNHISWLNFVSFNWITQLLRCLK-NDDFVLPCIEETSSIEHYSTNLN :   64
                           k         G         S   F tF WIT   n        F LP i    I yY   L

                  *       100         *       120         *       140         *       160
pbMRP  : KNLKVFRKKKKSFISRFFSKILSFTVNLKKSDRNKKNRNRIKDDDYFYEYN----RGIIAALTYTFKQPVLIISLLYILH :  144
pyMRP  : KNLKVFRKKKKSFLSQIFSKIFSFIVKKKNDRNKKNDR---IEDEDYYEYN----RGLISALAYTFIQPVLIISLLYIFH :  136
pkMRP  : NNLRGFKKRRRKNDNSTVNS-NEGSHKSSLNGMSN--TETSYSLQKR---------GIIFALLKTFKNQLSCITFFYVIH :  136
pvMRP2 : RHLKGFKKRKRPNGSGTQNNPSERPHGSTVQGTSNEESHYYCPYQKR---------GITLALLRTFRKQLSCITFFYVIH :  148
pfMRP2 : RNVRNIQLRKYNKYNKYKNYCCSKYNDGNKSCSCSTKNHTNKFHKRKKEDHFLKSSGITYAVLKTFKYYLSLISFFHIIH :  144
          nl  f k k                                              Gi  Al  TF      I   y  H

                  *       180         *       200         *       220         *       240
pbMRP  : ALFLVFVAICIEKYISIIKG-HHVFSPLLQSKTAKLLSAFVLIMVLSLNLFMDSAVSYIHNKLIIDMEVTVMHFLYKINM :  223
pyMRP  : ALYLVFVAICIEKYISIIKG-HYVFSPLLQFKTAKLLSAFVLIIVLSLNLFLDSIVSYMHSKLIIDMEVTVMHFLYKINM :  215
pkMRP  : TLYLIFVALCIENYILLIRG-EGNPWIPFLRKHKEIAFGLFLIGVICFDLFFDAILTFFDYRLRLNMEITLMYFLYKINL :  215
pvMRP2 : TLFLIFVALCIENYILLLKG-QGKVWLPFAHKYKELAFGFILVGVICVDVFFDAVLTFFDYRLRLNMEITLMYFLYKINL :  227
pfMRP2 : TIFLIFVAVCIEKYVLLIKGGSNVVTLPFGFKNSKVLFGFIVISVIFISQFFDALLCYYDFRLRVNMEVTVMYFLYKITL :  224
          l L FVA CIE Yi  ikG           K       f li V     F D         L   ME T M FLYKIn

                  *       260         *       280         *       300         *       320
pbMRP  : GVFNHCILN------------------QYHDDNNNN---LHEISNNNSENSTCANIDSYKNNTHGTSKNYIIKVENEDEN :  282
pyMRP  : GIFNHCILS------------------PYHDNNNNNNNNLLEMSNNNSENSTCTNVDYYKNNTR--------DDISKNCI :  269
pkMRP  : ENFRGTLSSSPYIALGDMEDTSPMLKGEEDSPSPEMNRPEGETKTGDDHSVKVEGVCSDRLGGDPITCSSIGCQAIPCDP :  295
pvMRP2 : EDFRGKLCSFPYPEG-----------GAGAQDAVPMLTGGGAREGGDHHDVQVEGAASAGG---ALSCPPFACQPAACDP :  293
pfMRP2 : GNFNNQLINRNDIYDDHSEEGKGQHKNQCQYDENDQNDQNEQSDLRDIRHMHDKHVKHNDEESKDSTNSTTYIKNNNNQM :  304
           F

                  *       340         *       360         *       380         *       400
pbMRP  : AEYKNETNQSNANTTISTNNLTDFSK------------------------------------KNDLNNKLE-DNENVIEH :  325
pyMRP  : IKVENETSQSNANTTISTNNLTNFSK------------------------------------KFDLNNKLEEDNENVIEN :  313
pkMRP  : IGCEAVSGEPIVIDTVVEEKGSVGSKGEVKISDDHLGGESKEKTIKREILSPRVEVDSTKVNGESNEYPDEAVQEEDDKR :  375
pvMRP2 : RGCVGEKQDVPSGEAASSVAAVRGG------------------AAPAEEAKPKGEIVQREESSTALPDDPPGEWAEGDKE :  355
pfMRP2 : SHINDLSITNNMSDVHILSSIKNQDQ-------NNSNNVSSMDSNTNYMSKTTCLTCTGSEFNKEEKDEKKELLEEAKKK :  377
                                                                                     e

                  *       420         *       440         *       460         *       480
pbMRP  : NNGDGGINIYNIMFIDTPSLIYFISSAIIANGMLIKLVISFYMFYHKMGKDSIIIGIFLVLLLYGIILLCELISSMLKKK :  405
pyMRP  : NKEDGDINIYNIMFIDTPSLIYFISSAIMANGMFIKLVISFYMFYHKMGKNSIIIGIFLVLLLYGIIFLCEFISSMLKKK :  393
pkMRP  : DNDSCDINIYNIMFVDTPFLIYFISSIIDFCNMVIKFTISFYMFYYKMGREAVVNGILLIAFLYSLMFAFELASSVFKIR :  455
```

```
pvMRP2 : ESDSCDINIYNIMFVDTPFLIHFISSMIDLCNMLIKFTISFYMFYFKMGREAVVNGILLIIFLYSLMFAFELASSLFKIK :  435
pfMRP2 : DKEVFDISIYNIMFIDTPFLIYFITALIELANMIIKFIMSFYMFYYKMGSAAVLNGALLIIIMYGLMFAFEFSSSLFKLK :  457
              dInIYNIMF DTP LIyFIss I    M IK  iSFYMFY KMG      Gi L   lY   f  E  SS  K k

                  *       500         *       520         *       540         *       560
pbMRP  : YLKYQDKRIDNMNHVLKEYKLMKMFNWESIAFDYVNKFREKELKYCKYRIYLSSISNYINAISVHCVEIVIFFVYIRNKL :  485
pyMRP  : YLKYQDKRIDNMNHVLKEYKLMKMFNWESIAFDYVNKFREKELKYCKYRIYLSSLSNYVNAISVHCVEIVIFFVYIKNKL :  473
pkMRP  : HLRCRDSRINNMHHILKEYKLMKMFNWESIAFDYVNRHRKKEMKVCTIRIYLNSLSNYINAISMNVVEVAIFFIFIRGEL :  535
pvMRP2 : HLKCRDARISNMHHILKEYKLMKMFNWEAIAFDYVNRHRKKEMKFCTVRIYLGSLSNYINNISMNVVEVAIFFFFIKGEL :  515
pfMRP2 : YLKYRDTRISNMHHILKEFKLMKIFNWESIAFDYVNMFRIKEMKICKIRTYLSSLSNYVNNISVNIVEVAIFFFYIRSEL :  537
          Lk  D RI NM H LKEyKLMKmFNWEsIAFDYVN  R KE K C  RiYL SlSNY N IS   VE  IFF  I   L

                  *       580         *       600         *       620         *       640
pbMRP  : NNNDQIDVNSVITPLFVYKSLINGIVSFPTIFNNLLEGPISNDRVNKYINHYFHDNISSKLFYSKIKNEKKNSHLKKK-- :  563
pyMRP  : NNNDHIDVNSVITPLFVYKSLINGIVSFPTIFNNLLEGPISNARVNKYVKHYFHDNISSKLFYSKIKNEKTNSYFKKKKN :  553
pkMRP  : NSNKPINVSSIITPLFLYKSLIAGMSNLPNIINNLLEGAINIGRINRYIEHYMFQ----------FDREDRNGYCTYRND :  605
pvMRP2 : NSNKPINVSSIITPLFVYKSLIAGMSNFPNIINNLLEGTINIGRINKYIRHYMFR----------SEGSEAGGYDHRIGL :  585
pfMRP2 : KSNKTVSFSSLITPLFVYKSLISGVSNFPNIINNLIEGAINIKRINKYINYYLFNNDMNDYFKNSLNGMKSNTCNFQNGN :  617
         n N  i v S ITPLFvYKSLI G   fP I NNLlEG I   R NkYi hY                   n

                  *       660         *       680         *       700         *       720
pbMRP  : -----------------------------NNNYMNMQGKSEKIIPSKKNTFYTSFLNMFSNDDYYYSSN----------- :  603
pyMRP  : NNSYISDKKNNNSYISGKKNNNSYIFDKKNNNYMNMQGKSEEIIPPKKNTFYTSFLNMISNDNYGYSYDDNNDDNNNDDN :  633
pkMRP  : --------------------HKIGIQTCSTFGRHNKMKSNSNKHCDDSNWRSGFYKYFFFNKGYGMPGKGKSNTNGYYYR :  665
pvMRP2 : --------------------HPG---AAPAAGSGDRGGKRNNQHGEDPHWG-ALIKSIFFGKGYGKAGKARKAG-KAAKS :  640
pfMRP2 : --------------------TNNVNGYVDDYVDDYVDDYVDDYVNDYVNDYVDDYMDHMMEYNININSKNGCSSKSRKNN :  677
                                                         n              y

                  *       740         *       760         *       780         *       800
pbMRP  : ---YNDHGSNTTNFEWENENNKIYRNNSGDHINNKIVTIDILNKNSKMNEINEGIKSKNILDEKYGIDKKTIIKLENCYY :  680
pyMRP  : NNDNNNHGGNAANIEWESENNKIWRDNGDDNNNSEIVTIDILNKNGKMNKINKSIKSQNIFDEKYGIDKNTIIKFENCNF :  713
pkMRP  : SEKGNDALGGVVAEKVTGRLSGVSIGGGKGHQASDVILKMNGCYFSPDKSPTGKGRHSRVDSNQ---------------- :  729
pvMRP2 : GGSNRTHYGAARGGLAAGTAVEVAAGKEAAPPPGEVILKLSGCYFSAEKPPSGGHDATGDATHQPNGQPN---------- :  710
pfMRP2 : KSFSKDHFSNHEQGTMQSFYKFLDTHKNKTKQKSDCKKKCSNNQLSKSNNNNVVGESNNSLFQDRKGWYPKNKHFKDNIV :  757
               h                                       s

                  *       820         *       840         *       860         *       880
pbMRP  : KPVKFEN-NYNQSKNMKLKNVNFTLKNNTIAIIIGDIGSGETLFFNSILGKFKLLNGNYYIKNFIY-DMPVLYAPQINWL :  758
pyMRP  : KPVKFEN-NYNQSKNIKLKNVNFTLKNNTIAIIIGDIGSGKTLFFNSILGKFKLLNGNYYIKNFIY-DMPVLYAPQINWL :  791
pkMRP  : -------------TDALLKNVNLTLKNNTLVVILGNVGSGKTLFFNSLFGKLKLSQGNCYIKNFVH-DMPVMYVPQFYWV :  795
pvMRP2 : --------GQPLVPAALLKNVNLTLRNNTLVVILGNVGSGKTLFFNSLLGQLRLSRGSCYVKSFAN-DTPVMYVPQFYWV :  781
pfMRP2 : INMKNCYFSSKNNDDYILKNINLTLKNNSVVIILGNVGSGKTIFFYSLLGQFKLSCGSFYLKNYIYKYMPILYVPQFNWI :  837
                          LKNvN TLkNNt   I G  GSGkTlFFnS lG  kL  G  Y Knf   dmPv Y PQ   W

                  *       900         *       920         *       940         *       960
```

```
pbMRP  : SDGTIRSMITFENEFDPYIYYLAISQSELINDIYSFKNLDMRYVNDEHSLSKGQKSRISLARSLYYHYINMKQLRTEYIE :   838
pyMRP  : SDGTIRSMITFENKFDPYIYYLAISQSELINDIYSFKNLDMRYVNDEHSLSKGQKSRISLARSLYYHYINMKQLCTENIE :   871
pkMRP  : TIGTIRSMIIFGNRFDPYLYYRAIVQSELINDMNTFKKKDLRYVGDEHSLSKGQKARICLARALYHHYIHMSDLLIDYEK :   875
pvMRP2 : TIGTIRSMILFGNRFDPFLYYRAIVQSELVNDMNTFKRKDLRYVNDEHSLSKGQKARICLARALYHHYIHMSDLLVDYER :   861
pfMRP2 : SLGTIRSMILFGNKYDESIYYDVIVKSELFHDIISFKKKDMRYISDEHSLSKGQKARICLARALYHHYIHMKHMNLYYQK :   917
           GTIRSMI F N fDp  YY aI qSEL nD   FK  D RYv DEHSLSKGQK RI LAR LY HYI M  l   y

                  *       980         *      1000         *      1020         *      1040
pbMRP  : MKNAEESTFDS-------------------------------TKESNFGLNKFATNYYDKNIDNMKSVRISKSIKNFTLH :   887
pyMRP  : MANVEESTFNN-------------------------------THESNLGLNKFTTNYYDKNIDNMKSVRFSESVQNFTLH :   920
pkMRP  : DIRINKEWREKVMLEKLYGTDNSSCKEPSGVRSDSSMGHHEREKGKNILIKGEKVGKKKEGTYDEPLSQEEKYQLHDAYH :   955
pvMRP2 : DVRVNKKWRERGPSEGLFKTASSLSRGRSGGSNGSRSHPKEEKRKKLFVKGGDADQGGKKGTDDEPSSQEEKYELHDVDN :   941
pfMRP2 : NELIN-------------------------------------EKMKKISLKRDDNHTAQRSNDNTPNNNNTDNNNTSDNN :   960
                                                                       d

                  *      1060         *      1080         *      1100         *      1120
pbMRP  : NDNQFENSINPNIYNDESEQIKGNNKNSKSDTN-----------------SFISDCKFSYPDLQFLMNNNYLKDCLEKND :   950
pyMRP  : NGDQFENSMSSNICNGKIEQIEEGDKNDKGGKSD----------KIDSNSSFTSDRNFSYSDLQFLINNNYLKECLDKNN :   990
pkMRP  : STNDTLNETNMINASLHGSPVKSGKYRKEEQEG----DQKQNGRGSNGHVIIGPTGEMYTENNNLFKKNSLLKECLEQEQ :  1031
pvMRP2 : STNYTFNTANMTNTCSHVSRVKNARGEEEEDDEELHPTQQQRRGGSNNDVIIGPSGEMYTQNCDLFKNNSLLKECLEQGR :  1021
pfMRP2 : NTSNNNNTSDNNNTSDNNNTSDNNNTSNNNNTD---------------------------NNNTSNNKNSCSKNLTEERN :  1013
               N                                                             N  lK cle

                  *      1140         *      1160         *      1180         *      1200
pbMRP  : MSYLYLLDDIFTSLDPYISRNIFYNLFCDKEKLKIIKNHCGIVITINENAFNSFIMKDIIE-NIQYNVDIYKLENGSLDF :  1029
pyMRP  : MSYLYLLDDIFTSLDPYISRNIFYNLFCDKDKLKNIKNHCGIVITINENSFNSFIMKDIIE-NLQYNVDIYKLENGSLYF :  1069
pkMRP  : MSYLYLLDDLFCALDPCISKNIFYNLFCSNDDVEGFKRNCAFVLTANQNIWNSFLDEDIIR-QLQYGVEIYRLEDRTLVY :  1110
pvMRP2 : VSYLYLLDDLFCALDPCISKNIFYNLFCSRENVEGFKGNCGFVLTANQNIWHSFLEEDIVR-DLQYDVQIYRLQNRSLVY :  1100
pfMRP2 : ISYLYLFDDLFTSLDPCISKDIFYNLFCDKEKIQHFKKNSSFILSISEIILNSFISSNCILNNMQYDVLIYKLENSTLHY :  1093
          SYLYLlDD F  LDP IS nIFYNLFC        K  c  v t n n  nSF    dii    QY V IY Len  L

                  *      1220         *      1240         *      1260         *      1280
pbMRP  : QGNINEYIQKKNIQIKASDVIIKKETENEKREKMLSFFKKTGISMNCSKMSDQVFYMSMSLELCYQNNPQTEVEITNSDN :  1109
pyMRP  : EGNINEYIQKKNIEIKISDVIIKKETENEKREKMFSFFKKTGISMKDSKKNDQVFYMSMSLELCHQNNPQTQVEIADNDN :  1149
pkMRP  : EGDIHTYMKNNGIVAQRGKGSKEGSSPIAPVKGNTNVSASQTASSGEAATGSSSSSSTSRMLDFFSEQQSGRKYSHKNDM :  1190
pvMRP2 : EGDVRAYMRKQGIAPGAASGAVEAAN-----AANTANAANTAAATPAAATATPAPHSTSQMLEFFSEQQGGGKNALKNDL :  1175
pfMRP2 : EGNLVDYIKKNNIVVKEDIVQTNKQCEKKSLTNEQVKSMLSLNEDWNYMHRVKKKSITQKETTKNYDNNNDNNNDNNNDN :  1173
         eG    Y  k  I                                                                 nD

                  *      1300         *      1320         *      1340         *      1360
pbMRP  : IESSLSTSVIYKRDNSYFDKNNIIYNKIILLKELKLLHHIKAEANNETPNINKKFTILMNNHFNKIVNESILKDRIHNIS :  1189
pyMRP  : IENPSNITVVYKKDNSHFDKNNVIYNKIILXKELKLLHHLKSEANNEMPNLNKKFTILMNNHFNKIVNESILKDKNNNIS :  1229
pkMRP  : KFQKFVALKELKSTYSCRIEPLDSPSVHEFGRKYTTVTQNYVEMPNGYITNGYITNGYITNAMRNVSISSIGSLNKTMIN :  1270
pvMRP2 : KYQKFVMLKELKSTYSCRLEPLDSASAQGFGRKYTTVTQNYVEKRDEHVMN---------NTIGSVSISSLGSTRKTTIS :  1246
pfMRP2 : NNDNNNDNNNDNNNDNNNDNNNNNNNNNVNVSKEILSCEIKTQDHYNNIHCSKTNFEKHNNSIYSKKENETRKIHSGDIK :  1253
```

```
                   k   s                            e                  N        s        I

                   *       1380          *        1400          *        1420          *        1440
pbMRP  : TYEMAKIKKDQIRKYIGNFIVDTKGNNEMKCLKNK--------------------------------------------- : 1224
pyMRP  : TDEIAKIKKDEIRKYIGNFIINKKENNEIKCLKNK--------------------------------------------- : 1264
pkMRP  : SYAEVLIKEKKNVEGVGNDNRRNRSFDEDD-------------------------------------------------- : 1300
pvMRP2 : NYAEVLVKEKK---SRTRGNSCSLSFEQDD-------------------------------------------------- : 1273
pfMRP2 : YHKFMVLKQFKTIYSFKTIYSFNTEETNDDEYNKTYYRKYTKVIQNYDNHCLEGKKKSFRNYKSINSYNEILIKENMKVW : 1333
                K

                   *       1460          *        1480          *        1500          *        1520
pbMRP  : -------KTHINEFEEINAMIKKNLKEHYTYYNNYIRYNSNNDTNIDVARKGNINFETFRWYFRSIGNAIIICIIIFIIF : 1297
pyMRP  : -------KTDVEEFEKINAMIKKNLKEHYTYYNNYIRDNSNNDTNKDVARKGNIKFETFKWYFKNVGNVIIICIIIFIIF : 1337
pkMRP  : -------KITLHEFEKVSKVLQAKLKQNYIYEYMGNEDNGDEGEEEELRFKGNIKWETFAWYLPMIGTPLIAVILFFMVI : 1373
pvMRP2 : -------KITLHEFEKVSKVLQAQLKDNYMYEYLGNQENGDEGEEEELRFKGNIKWETFVWYLRMIGNSLIAVILLFMIV : 1346
pfMRP2 : EDDTYYGNDYIDEYEKIKKNVLSKLKYNYYISCDYNNSDHFDFN-EELKFKGNIKLETFWWYLKKIGRPLIIVIIIFMLL : 1412
                k    EfEk    l   LK  Y y     n            KGNIk ETF WY   iG   I  I  F

                   *       1540          *        1560          *        1580          *        1600
pbMRP  : SIFLDEVKNMILFLVSAILKTKD-KSYEEIIQTKLVYLKYFILLPALSLVTTFISYMVIAHGIMISAKAIHTEVFKSMLY : 1376
pyMRP  : SIFLDEIKNMILFLVSAILKTKD-KSYEEIIQTKLVYLKYFIILPSLSLITTFISYMVIAHGIMISARKMHTEVFKSMLY : 1416
pkMRP  : SIFTDEIKNMILFMASTIIKSKG-QRESEILEKQLVYMKWFVLLPCVSLVTHLTSFMLIAHGIAISAVKVHTEVLMSILY : 1452
pvMRP2 : SIFTDEIKNMILFMASTLIKSEEEQNDSEILKKQLVYLKYFVLLPCVSLVTSLIAFMLIAHGIAISAVKVHTQVLQSILH : 1426
pfMRP2 : SIFTDEIKNLILFLASTILKSGD-KKDEEILNQQLVYLNYFILLPSISLLTILISFMLIAHGIVKSAIKVHTEVLLSILY : 1491
         SIF DEiKNmlLF  S l K         EI    LVYlkyF lLP  SL Tt is M IAHGI iSA k HTeV   S Ly

                   *       1620          *        1640          *        1660          *        1680
pbMRP  : APIPAFYSHNIGNIINRFIIDTHTLDNGIIKRCYKSFFTVSKFISTVILLIFMFKKTYIMLPFITLIVYYGIFKKYSLAC : 1456
pyMRP  : APIHAFYSHNIGNIINRFIIDTHTLDNGIIKRCYKTFFTISKFISTVILLIFMFKKTYIMLPFITIIVYYGIFKKYSVAC : 1496
pkMRP  : APIHAFYSNNLGNIINRFITDINVLDNGIIKRIYKTFYTFFRFLFTIYLLNYMVYQTIYVLPIIIFLIYVCVFQRYSRGC : 1532
pvMRP2 : APIHAFYSNNLGNIINRFITDINVLDNGIIKRIYKTFYTFFRFLFTIFLLNYMVYQTVYVFPMITFLIYVCVFQKYSRGC : 1506
pfMRP2 : APIHAFYSNNLGNIINKFITDVNILDNGIIKRIYKSFYTLFRFLFTLFLLIYMVKYTIVIFPFIMLIIYFFVFNKYSKGC : 1571
         APIhAFYS N GNIINrFI Di  LDNGIIKR YK F T   F  T  LL  M   T     P Ii   Y   F kYS  C

                   *       1700          *        1720          *        1740          *        1760
pbMRP  : KEAQRGYLCSHSPICSIFSNTIHGKDIINLYKKNYCILKKFENSIYALRNFTLFKWGITAWASLYIQLVSLCLTSFYILY : 1536
pyMRP  : KEAQRGYLCSHSPICSIFSNTINGKDIINLYKKNDLILKKFENSIYALRNFTLFKWGITVWASLYIQLVGLCLTSFYILY : 1576
pkMRP  : KEAQRGYLSAHAPLCTIYSNTILGKEIIYLYGKSKHFLDLYSKGVFNYKNYTIFKWCLTIWASLYVQLIVLCLTSFYIIY : 1612
pvMRP2 : KEAQRGYLSSHAPLCTIYSNTIMGKDIINLYKKNQHFLDLYAKRVFDFKNYTIFKWCLTIWASLYVQLIVLCLTGFYIIY : 1586
pfMRP2 : KEAQRGFLASHAPLCTIYSNTIIGKLVINLYKKNNYFLTLYKQKIFDFRNYTIFKWSITIWASLYVQLIVLALTFFYIIY : 1651
         KEAQRGyL sH P C I SNTI GKdiInLYkKn    L             N T FKW  T WASLY QL  LcLT FYI Y

                   *       1780          *        1800          *        1820          *        1840
pbMRP  : PHVFSIFKNSSEHIDIDVDDYISYSEVIGYCITFSCSLGYIIKSFLYDYTHVEKEMCSIQSIQELSKIKNISDETSFT-- : 1614
pyMRP  : PHFFSIFKNSPEHIDIDVDNYISYSEVIGYCITFSCSLGYIIKSFLYDYTHVEKEMCSIQRLEELSKIKNINDEASAS-- : 1654
```

```
pkMRP  : PYVLEPYLGDK--KDPHFMNEEKNANTIGYCITFSCSLGFIIKSLLYDYTHVEKEMCSTQRLEECSQMFKEQVNYDD--- : 1687
pvMRP2 : PYVLHPYLGNK--QDPNFMNKEKNASTIGYCITFSCSLGFIIKALLYDYTHVEKEMCSTQRLEECSQMINEQVGYAD--- : 1661
pfMRP2 : P---HFFFKHA--KQDHEINYEKEASTIGYCITFSCSLGFVIKSLLYDYTHVEKEMCSTQRLEECSKMIKDEGYSDDNIT : 1726
         P             d    n       IGYCITFSCSLG iIKs LYDYTHVEKEMCS QrLeE S

                  *      1860         *      1880         *      1900         *      1920
pbMRP  : ----------------------------------------------------QIENHEYSYENNKNVENANYLNNKTEDG : 1642
pyMRP  : ----------------------------------------------------HVG-----HFENGKSKKFEKFENGENVN : 1677
pkMRP  : --------------------------KEG-----------------------------MLPTQMLTMQTASTQVPVVKDT : 1712
pvMRP2 : --------------------------EGGEAAKCGKGGPPPWEEVSREVPKEVSKEVSTEVSTEVSKEVLKEVPKEVPLT : 1715
pfMRP2 : LQNNDPTHEDNNNNNNNNNNEQDANLFKSVPGSYDPNDKENMRKYKTEIVNNTKDMYSIINKDDMLTHSINNKNNKLKKL : 1806

                  *      1940         *      1960         *      1980         *      2000
pbMRP  : ILITPSQFPKSKYGLEFKNVYVSYKKKVYIDKLKNIYYYANEKSCLRNINFYALKSQNIGIIGKSGAGKSTIVMAILGLI : 1722
pyMRP  : ALVSPTQLPQSKYGLEFKNVYVSYKKKVYIDKLKNIYYYANEKSCLRNINFYALKSQNIGIIGKSGAGKSTIVMAILGLI : 1757
pkMRP  : PPMCAPENGKAKYGLHFENVFVSYKKKIFIDRSRNLYYYANEKSCLRNLNLYALKGQKIGIVGRSGAGKSTTILSVLGLI : 1792
pvMRP2 : SPSKDVPCGKAKYGLHFENVFVSYKKKIFIDKSRNLYYYANEKSCLRNMNIYALKGQKIGIVGKSGAGKSTTILSVLGLI : 1795
pfMRP2 : YTSPHIDINKIKYGICFERVFVSYKKKICVDRKNNKYEYVNEKSCLKNINIYALKNQKIGIVGKSGAGKSTMILSILGLI : 1886
                  k KYGl F nV VSYKKK  iD   N YyYaNEKSCLrN N YALK Q IGI GkSGAGKST     LGLI

                  *      2020         *      2040         *      2060         *      2080
pbMRP  : STSKGEIKIDGRDIRSIPLNEKKKIIGILPQSSFVFSHWNIRTYIDPYQKFSDNEIIDAFETIGINLTCADLNKYIYKTK : 1802
pyMRP  : STSKGEIKIDGIDIKNIPLDEKKKIIGILPQSSFVFSHWNIRTYIDPYHKFSDNEIIDAFETIGINLTCADLNKYIYKTK : 1837
pkMRP  : PTTRGTISIEGRDIKTMTLEERKNTIGLLPQSSFVFFHWNIRTYIDPYKNFTDNEIMDAFKLIGINLQMEDLYKYIYKQK : 1872
pvMRP2 : PTTRGTISIEGRDIKTMSLQEKKRIIGLLPQSSFVFFHWNIRTYIDPYGSFSDDDIMEAFKLIGINLRKEDLYKYIYKQK : 1875
pfMRP2 : GTTRGRITIEGQDIKTLTLDERKNMIGVLPQSSFVFFHWNIRTFIDPYKDFTDDEIVDAFKLIGINLSYDDLDKYIYKQQ : 1966
          T  G I I G DIk   L E K  IG LPQSSFVF HWNIRTyIDPY  F D eI dAF  IGINL   DL KYIYK k

                  *      2100         *      2120         *      2140         *      2160
pbMRP  : KKMN-----------EYDKSKYTKTNKKYNENYILMSDDSIRYLSLVRIYLNRNNYKLLLIDEIPVVNFN-KNNSEFNNF : 1870
pyMRP  : KKKNN----------EYHKSKYTKINKKNNENYILMSDDSIRYLSLVRIYLNRKNYKLLLIDEIPVVNFN-KNNSEINNF : 1906
pkMRP  : KRAS----QDGRRVYNGRDKFDKSKSSSTVANSLSLSDDCIRYLALVRIFLNRHNYKLVLIDEIPVLNLN-LSNSNTNNF : 1947
pvMRP2 : KKSASRDDRAGGGLPNGADKGQKGNSSSSAANFLAVSDDCIRYLALVRIFLNRHTYKLVLIDEIPVLNLN-LSNSNANNF : 1954
pfMRP2 : RQQQ-------KKKNKNTHNLWKKKSFIDLTNSISLSDECIRYLSLVRLFLNRHKYKLILIDEIPVLNFC-YNTKKLTNF : 2038
         k                              N    SDd IRYL LVRi LNR  YKL LIDEIPV N n   ns  nNF

                  *      2180         *      2200         *      2220         *
pbMRP  : FTKNLKPFDYIIENYFKHITILIISHDTRTLSSCDFICVVSKGEIVYKCNYSDVETQTQLANIIQDQAN- : 1939
pyMRP  : FTKNLKPFDYIIENYFKHITVLIISHDTRTLSSCDFICVVSKGEVVYKCNYSDVETQTQLANIIQDQAN- : 1975
pkMRP  : FSSDVKSFEYIIRNYFSHITVLIIAHDVSTLSCCDFIYVVAKGEVSYKCSYSDIKTQAALAALIQKQTE- : 2016
pvMRP2 : FSSDVKSFEYIIRNYFAHITVLIIAHDASTLSCCDFIYVVAKGEVSYKCSYADIQTQAALAALIQQQTE- : 2023
pfMRP2 : FTTDIKSFDYIIRTFFQNTTVLIIAHDASTLSCCDFIYVIAKGEVVYKCSYKDVKTQTELANLLQEKQLN : 2108
         F    K F YII nyF hiTvLII HD  TLS CDFI Vv KGEv YKC Y D  TQ  LA  iQ q
```
